# Practice of standardization of CLSI M45 A3 antimicrobial susceptibility testing of Infrequently Isolated or Fastidious Bacteria strains isolated from blood specimens in Guangdong Province 2017–2021

**DOI:** 10.3389/fmicb.2024.1335169

**Published:** 2024-04-29

**Authors:** Nanhao He, Xu Yang, Anwarul Haque, Jiakang Chen, Yingyi Guo, Jiahui Li, Likang Yao, Chuyue Zhuo, Jiong Wang, Yijing Wang, Mingxin Li, Yexin Lin, Shunian Xiao, Chao Zhuo

**Affiliations:** ^1^Department of Guangdong Antimicrobial Resistant Bacteria Monitoring and Quality Control Center, The First Affiliated Hospital of Guangzhou Medical University, Guangzhou, China; ^2^Department of Infectious Diseases, School of Medicine, International University of Health and Welfare, Narita, Japan; ^3^Guangzhou Institute of Respiratory Health, The First Affiliated Hospital of Guangzhou Medical University, Guangzhou Laboratory, Guangzhou Medical University, Guangzhou, China

**Keywords:** bloodstream infections, clinical laboratories, CLSI M45 A3, Infrequently Isolated or Fastidious Bacteria, standardization

## Abstract

The concentration of antimicrobial agents in environments like water and food has increased rapidly, which led to a rapid increase in antimicrobial resistance levels in the environment. Monitoring of bacterial resistance levels is considered as a necessary means to control the bacterial resistance. Reference standards are critical for antimicrobial susceptibility testing. CLSI M45 A3 standard defines pathogenic microorganisms that cause infections less frequently than those covered by CLSI M02, M07, and M100 as Infrequently Isolated or Fastidious Bacteria and specifies antimicrobial susceptibility testing methods. Our study investigated the epidemiology and antimicrobial susceptibility testing data of Infrequently Isolated or Fastidious Bacteria strains isolated from blood specimens in 70 hospitals in Guangdong Province between 2017 and 2021. We defined testing methods other than those specified in CLSI M45 A3 as “Non-Standardized.” The proportion of standardized antimicrobial susceptibility testing for penicillin increased significantly (*Corynebacterium* spp. 17.4% vs. 50.0% *p* < 0.05; *Micrococcus* spp. 50.0% vs. 77.8% *p* < 0.05; *Abiotrophia* spp. and *Granulicatella* spp. 21.4% vs. 90.9% *p* < 0.001), while for cefotaxime (*Corynebacterium* spp. 0.0% vs. 45.2% *p* < 0.05; *Abiotrophia* spp. and *Granulicatella* spp. 0.0% vs. 14.3% *p* = 0.515) and vancomycin increased finitely. Non-standardized methods were used for all other antimicrobials. Due to limitations in the economic and medical environment, some clinical laboratories are unable to fully comply with CLSI M45 A3 standard. We recommend that CLSI should add breakpoints for disk diffusion method to improve the standardization of antimicrobial susceptibility testing.

## Introduction

Microorganisms added to the standard CLSI M45 that causes infection less frequently than those covered by CLSI M02, M07, and M100 are defined as “Infrequently Isolated” or “Fastidious Bacteria.” According to CLSI M45, “Infrequently Isolated” or “Fastidious Bacteria” include coryneform bacteria, *Bacillus* spp., *Granulicatella* spp., *Aeromonas* spp. and potential bacterial agents of bioterrorism, etc.

These bacteria are widely distributed in the environment and food, and can affect human health. For example, *Aeromonas* spp. are distributed in aquatic environments (Majeed et al., 2023), and the pathogenic bacteria *Bacillus* spp. (Özdemir and Arslan, 2019) and *Micrococcus* spp. (Tizabi and Hill, 2023) in food. Due to the widespread use of antimicrobial drugs, antimicrobial resistance levels are increasing worldwide (World Health Organization, 2022). It is worth noting that as the concentration of antibacterial drugs increases in environments such as in water and in agriculture farms, multi-drug-resistant (MDR: resistant to ≥ one agent in ≥3 antimicrobial classes) genes are emerging in Infrequently Isolated or Fastidious Bacteria (Zhang et al., 2022; Yang and Wu, 2023). Many reports have demonstrated that antimicrobial-resistant Infrequently Isolated or Fastidious Bacteria are increasingly occurring in the environment (Gonzalez-Avila et al., 2021; Algammal et al., 2022a; Wu et al., 2023). Therefore, we believe that it is necessary to monitor the resistance levels of Infrequently Isolated or Fastidious Bacteria isolated from clinical patients. The CLSI M45 standard defines methods for susceptibility testing and interpretive criteria for Infrequently Isolated or Fastidious Bacteria is an important reference standard for clinical microbiological testing.

However, antimicrobial susceptibility testing standardization of Infrequently Isolated or Fastidious Bacteria in clinical work has not been fully studied. This study collected Infrequently Isolated or Fastidious Bacteria isolated from blood samples from 70 hospitals in Guangdong Province, and analyzed the epidemiology and antimicrobial susceptibility of Infrequently Isolated or Fastidious Bacteria. This study provided a basis for standardized testing of Infrequently Isolated or Fastidious Bacteria in clinical laboratories and provided data for monitoring resistance levels of Infrequently Isolated or Fastidious Bacteria.

## Methods

### Strains

The study included Infrequently Isolated or Fastidious Bacteria isolated from blood samples in 70 hospitals in Guangdong Province between 2017 and 2021. Identical strains isolated from the same site in the same patients were eliminated. Strains were identified using BD Phoenix M50, BD Phoenix 100 Incubator Bioreactor Colony Microbiology Culture, Sensititre ARIS HiQ AST, and VITEK^®^ MS IND MALDI TOF and VITEK^®^2 Compact. The definition of Infrequently Isolated or Fastidious Bacteria referred to CLSI M45 A3. According to the definition of CLSI M45 A3, *Bacillus anthracis* was not included in *Bacillus* spp. in this study. Potential Bacterial Agents of Bioterrorism included *Bacillus anthracis*, *Yersinia pestis*, *Burkholderia mallei*, *Burkholderia pseudomallei*, *Francisella tularensis* and *Brucella* spp. This study was approved by the ethical review committee of the First Affiliated Hospital of Guangzhou Medical University (GMU).

### Antimicrobial susceptibility testing

Referred to the antimicrobial susceptibility testing methods recommended by CLSI M45-A3 (2015) (CLSI, 2015) and the 2022 CLSI M100 standard (CLSI, 2022). The quality control bacteria for antimicrobial susceptibility testing were *Escherichia coli* ATCC25922, *Staphylococcus aureus* ATCC25923, and *Pseudomonas aeruginosa* ATCC27853.

### Susceptibility interpretation

The interpretation of antimicrobial susceptibility testing results of quality control bacteria referred to the 2022 CLSI M100 judgment standard (CLSI, 2022). CLSI M45 A3 recommends the broth microdilution method for antimicrobial susceptibility testing of Infrequently Isolated or Fastidious Bacteria, however, some hospitals used the disk diffusion method. Therefore, the antimicrobial susceptibility testing results in this study originated from both the broth microdilution method and the disk diffusion method. The results of the disk diffusion method were interpreted using WHONET 5.6 based on the 2022 CLSI M100 resistance breakpoints for common bacteria. The breakpoint standards for the disk diffusion method are the standards being used by the clinical laboratories of the hospitals, and the source is no longer available. Antimicrobial susceptibility testing results were not included when the number of strains analyzed were less than ten. The breakpoints of the broth microdilution method referred to CLSI M45 A3 and the 2022 CLSI M100 standard (CLSI, 2022). The breakpoints of the disc diffusion method and E-test method referred as follows (Supplementary Tables S1–S8).

### Data statistical analysis

WHONET 5.6 was used for data analysis. Data were processed through SPSS 26.0. The results were subjected to chi-square test, and the difference was considered statistically significant at *p* < 0.05.

## Results

### Epidemiological data

Our member hospital number has changed between 2017 and 2021, namely from 69 hospitals in 2017, 79 hospitals in 2018 to 70 hospitals from 2019 to 2021. The isolation rates of Infrequently Isolated or Fastidious Bacteria from 2017 to 2021 were 1.5% (401/27631), 1.4% (415/28858), 1.8% (530/29710), 1.7% (472/28134), 2.1% (694/32218), respectively, and a total of 2,512 strains were isolated. The isolation rate of Infrequently Isolated or Fastidious Bacteria in blood samples increased significantly between 2017 and 2021 (*p* < 0.0001). The proportion of Infrequently Isolated or Fastidious Bacteria isolated were, *Aeromonas* spp. 37.1% (933/2512), *Coryneberium* spp. 19.4% (488/2512), *Micrococcus* spp. 9.7% (244/2512), Potential Agents of Bioterrorism 6.7% (168/2512), *Abiotrophia* spp. and *Granulicatella* spp. 6.6% (165/2512), *Bacillus* spp. 5.7% (144/2512) and other Infrequently Isolated or Fastidious Bacteria 14.7% (370/2512). *Aeromonas* spp. was isolated in 98.5% (69/70) of the participant hospitals.

The proportion of isolation of *Aeromonas* spp. decreased significantly [44.4% (2017) vs. 27.5% (2021), *p* < 0.0001]. In particular, the proportions of isolation of *Aeromonas hydrophila* [28.2% (2017) vs. 16.0% (2021), *p* < 0.0001] and *Aeromonas sobria* [6.5% (2017) vs. 3.6% (2021), *p* < 0.05] among *Aeromonas* spp. decreased significantly. There was no significant difference in the proportions of *Aeromonas caviae* [6.2% (2017) vs. 5.0% (2021), *p* = 0.404] and other *Aeromonas* [3.5% (2017) vs. 2.9% (2021), *p* = 0.575].

The proportion of *Corynebacterium* spp. increased significantly during the study period [12.2% (2017) vs. 27.0% (2021), *p* < 0.0001] was isolated from 58.6% (41/70) of the hospitals. The proportion of *Corynebacterium striatum* [3.7% (2017) vs. 19.0% (2021), *p* < 0.0001], *Corynebacterium jeikeium* [0.5% (2017) vs. 2.0% (2021), *p* < 0.05] and *Corynebacterium afermentans* [0.3% (2017) vs. 2.3% (2021), *p* < 0.01] increased significantly. The proportion of other coryneform bacteria decreased significantly [7.7% (2017) vs. 3.6% (2021), *p* < 0.005].

*Micrococcus* spp. was isolated from 53.0% (37/70) of participant hospitals. The proportion of *Micrococcus* spp. decreased significantly [13.2% (2017) vs. 7.9% (2021), *p* < 0.01]. The proportion of *Micrococcus luteus* decreased significantly [11.2% (2017) vs. 6.9% (2021), *p* < 0.05], there was no significant difference for other Micrococci [2.0% (2017) vs. 1.0% (2021), *p* = 0.176].

*Brucella* spp. was isolated from 25.7% (18/70) of participant hospitals, and *Burkholderia pseudomallei* was isolated from 25.7% (18/70) of member hospitals. The proportion of Potential Bacterial Agents of Bioterrorism increased significantly [5.5% (2017) vs. 9.4% (2021), *p* < 0.05]. The proportion of *Burkholderia pseudomallei* [1.3% (2017) vs. 3.2% (2021), *p* < 0.05] increased significantly. There was no significant difference in *Brucella* spp. isolation [4.0% (2017) vs. 6.0% (2021), *p* = 0.142].

*Abiotrophia* spp. and *Granulicatella* spp. were isolated from 51.4% (36/70) of participant hospitals. There was no significant change in the proportions of *Abiotrophia* spp. and *Granulicatella* spp. [5.5% (2017) vs. 5.9% (2021), *p* = 0.773], including *Granulicatella adiacens* [3.5% (2017) vs. 4.0% (2021), *p* = 0.652], *Abiotrophia defectiva* [1.5% (2017) vs. 1.4% (2021), *p* = 0.941] and others [0.5% vs. 0.4% (2021), *p* = 1].

*Bacillus* spp. was isolated from 40.0% (28/70) participant hospitals. The proportion of *Bacillus* spp. increased significantly [4.0% (2017) vs. 7.6% (2021), *p* < 0.05]. The proportion of *Bacillus cereus* increased significantly [1.0% (2017) vs. 5.9% (2021), *p* < 0.0001]. The proportion of *Bacillus subtilis* decreased significantly [2.5% (2017) vs. 0.3% (2021), *p* < 0.005]. There was no significant change in the proportion of other *Bacillus* spp. [0.5% (2017) vs. 1.4% (2021), *p* = 0.254].

There was no significant change in the proportion of other Infrequently Isolated or Fastidious Bacteria [15.2% (2017) vs. 14.7% (2021), *p* = 0.818] (Supplementary Figure S1 and Supplementary Table S8).

### Antimicrobial susceptibility testing

#### *Aeromonas* spp.

Antimicrobial susceptibility testing was performed for 27.0% (252/933) of *Aeromonas* spp. isolates, 26.6% (153/575) of *Aeromonas hydrophila* isolates, and 26.9% (39/145) of *Aeromonas caviae* isolates.

In the antimicrobial susceptibility testing of *Aeromonas* spp. for cefotaxime, the resistance rate using broth microdilution method (93.7% 151/161) was 19.2%, the resistance rate using the disk diffusion method (6.2% 10/161) was 30.0%. All other antimicrobial susceptibility testing used the disk diffusion method. And in most cases, that was performed for a small number of isolates with resistance rates of 46.2% for cefuroxime, 46.2% for cefoxitin, 26.1% for ceftazidime, 34.1% for imipenem, 21.1% for aztreonam, 8.3% for amikacin, 4.3% for gentamycin, 0.0% for ciprofloxacin.

In the antimicrobial susceptibility testing of *Aeromonas hydrophila* for cefotaxime, the resistance rate using broth microdilution method (92.4% 85/92) was 17.6%. The resistance rate using the disk diffusion method (7.6% 7/92) was 42.9%.

All other antimicrobial susceptibility testing used the disk diffusion method, with resistance rates of 25.0% for ceftazidime, 27.3% for imipenem, 16.7% for aztreonam, 0.0% for gentamycin (Supplementary Table S9).

In the antimicrobial susceptibility testing of *Aeromonas caviae* to cefotaxime, the resistance rate using broth microdilution method was 33.3%.

#### *Corynebacterium* spp.

Antimicrobial susceptibility testing was performed for 84.1% (410/488) of *Corynebacterium* spp. isolates and 77.7% (206/265) of *Corynebacterium striatum* isolates.

In antimicrobial susceptibility testing of *Corynebacterium* spp. for penicillin, the resistance rate was 53.0% using the broth microdilution method (35.1% 115/328) and the resistance rate was 82.2% using the disk diffusion method (64.9% 213/328). In antimicrobial susceptibility testing for cefotaxime, the resistance rate was 70.0% using the broth microdilution method (25.9% 30/116) and the resistance rate was 58.1% using the disk diffusion method (74.1% 86/116). In antimicrobial susceptibility testing for vancomycin, the resistance rate using broth microdilution method (30.5% 120/394) and disk diffusion method (69.5% 274/394) was 0.0%. All other antimicrobial susceptibility testing used the disk diffusion method, with resistance rate of 26.7% for gentamycin, 67.8% for erythromycin, 84% for ciprofloxacin, 4.3% for doxycycline, 13.0% for tetracycline, 86.4% for clindamycin, 55.6% for trimethoprim-sulfamethoxazolel, 22.6% for rifampin. In the antimicrobial susceptibility testing of *Corynebacterium striatum* for penicillin, the resistance rate was 61.4% using the broth microdilution method (44.3% 70/158) and the resistance rate was 94.3% using the disk diffusion method (55.7% 88/158). In antimicrobial susceptibility testing for cefotaxime, the resistance rate was 88.9% using the broth microdilution method (36.0% 18/50) and the resistance rate was 81.2% using the disk diffusion method (64.0% 32/50). In antimicrobial susceptibility testing for vancomycin, the resistance rate was 0.0% using the broth microdilution method (38.8% 78/201) and the disk diffusion method (61.2% 123/201). All other antimicrobial susceptibility testing used the disk diffusion method. With resistance rate of 27.4% for gentamycin, 74.0% for erythromycin, 95.6% for ciprofloxacin, 14.1% for tetracycline, 92.6% for clindamycin, 59.3% for trimethoprim-sulfamethoxazole, 1.6% for rifampin (Supplementary Table S10).

#### *Micrococcus* spp.

Antimicrobial susceptibility testing was performed for 86.1% (210/244) of *Micrococcus* spp. isolates and 86.7% (189/218) of *Micrococcus luteus* isolates.

In antimicrobial susceptibility testing of *Micrococcus* spp. for penicillin, the resistance rate using the broth microdilution method (48.3% 72/149) was 18.1%, the resistance rate using disk diffusion method (51.7% 77/149) was 15.6%. In antimicrobial susceptibility testing for vancomycin, the resistance rate using broth microdilution method (45.6% 73/160) and disk diffusion method (54.4% 87/160) was 0.0%. All other antimicrobial susceptibility testing used the disk diffusion method, with resistance rate of 36.9% for erythromycin and 18.3% for clindamycin. In the antimicrobial susceptibility testing of *Micrococcus luteus* for penicillin, the resistance rate was 18.8% using the broth microdilution method (49.6% 64/129) and the resistance rate was 13.8% using the disk diffusion method (50.4% 65/129). In antimicrobial susceptibility testing for vancomycin, the resistance rate using broth microdilution method (47.4% 64/135) and disk diffusion method (52.6% 71/135) was 0.0%. All other antimicrobial susceptibility testing used the disk diffusion method, with resistance rates of 36.3% for erythromycin and 17.5% for clindamycin (Supplementary Table S11).

#### *Abiotrophia* spp. and *Granulicatella* spp.

Antimicrobial susceptibility testing was performed for 84.2% (139/165) of *Abiotrophia* spp. and *Granulicatella* spp. isolates and 83.2% (99/119) of *Granulicatella adiacens* isolates.

In the antimicrobial susceptibility testing of *Abiotrophia* spp. and *Granulicatella* spp. for penicillin, the resistance rate using the broth microdilution method (47.5% 47/99) was 2.1%, the resistance rate using the disk diffusion method (49.5% 49/99) was 44.9%, and the resistance rate using the E-test method (3.0% 3/99) was 0.0%. In antimicrobial susceptibility testing for cefotaxime, the resistance rate was 0.0% using the broth microdilution method (11.9% 7/59) and the resistance rate was 11.5% using the disk diffusion method (88.1% 52/59). In antimicrobial susceptibility testing for vancomycin, the resistance rate of broth microdilution method (13.2% 16/121), disk diffusion method (86.0% 104/121) and E-test method (0.8% 1/121) was 0.0%. All other antimicrobial susceptibility testing used the disk diffusion method, with resistance rates of 2.4% for ampicillin, 58.8% for erythromycin, 51.3% for clindamycin, 3.1% for chloramphenicol. In antimicrobial susceptibility testing of *Granulicatella adiacens* for penicillin, the resistance rate using the broth microdilution method (49.3% 35/71) was 2.9%, the resistance rate using the disk diffusion method (49.3% 35/71) was 45.7%, and the resistance rate using the E-test method (1.4% 1/71) was 0.0%. In antimicrobial susceptibility testing for cefotaxime, the resistance rate using the broth microdilution method (15.0% 6/40) was 0.0%, the resistance rate using the disk diffusion method (85.0% 34/40) was 14. 7%. In antimicrobial susceptibility testing for vancomycin, the resistance rate using broth microdilution method (11.4% 10/88) and disk diffusion method (84.1% 74/88) was 0.0%. All other antimicrobial susceptibility testing used the disk diffusion method, with the resistance rate of 3.8% for ampicillin, 59.0% for erythromycin, 56.2% for clindamycin, 4.5% for chloramphenicol (Supplementary Table S12).

#### *Bacillus* spp.

Antimicrobial susceptibility testing was performed for 72.2% (104/144) of *Bacillus* spp. isolates and 68.5% (63/92) of *Bacillus cereus* isolates.

In antimicrobial susceptibility testing of *Bacillus* spp. for penicillin, the resistance rate using the broth microdilution method (58.8% 47/80) was 38.3%, the resistance rate using the disk diffusion method (36.3% 29/80) was 86.2%, and the resistance rate using the E-test method (5.0% 4/80) was 0.0%. In antimicrobial susceptibility testing for vancomycin, the resistance rate using broth microdilution method (46.8% 44/94), disk diffusion method (48.9% 46/94) and E-test method (4.3% 4/94) was 0.0%. All other antimicrobial susceptibility testing used the disk diffusion method, with resistance rates of 81.8% for ampicillin, 71.4% for trimethoprim-sulfamethoxazole, 65.2% for rifampin. 3.6% for imipenem, 14.0% for erythromycin, 15.6% for clindamycin, 3.8% for tetracycline, 15.2% for ciprofloxacin, 3.8% for chloramphenicol, 0.0% for amikacin and gentamycin.

In the antimicrobial susceptibility testing of *Bacillus cereus* for penicillin, the resistance rate using the broth microdilution method (73.5% 36/49) was 88.9%, the resistance rate using the disk diffusion method (18.4% 9/49) was 100%, and the resistance rate using the E-test method (8.2% 4/49) was 0.0%. In antimicrobial susceptibility testing for vancomycin, the resistance rate using broth microdilution method (57.6% 34/59), disk diffusion method (35.6% 21/59) and E-test method (6.8% 4/59) was 0.0%. All other antimicrobial susceptibility testing used the disk diffusion method, with resistance rates of 78.6% for ampicillin, 61.5% for trimethoprim-sulfamethoxazole, 7.1% for imipenem.9.1% for erythromycin, 5.0% for clindamycin, 8.3% for tetracycline, 20.0% for ciprofloxacin, 0.0% for chloramphenicol, amikacin, and gentamycin (Supplementary Table S13).

### Potential bacterial agents of bioterrorism

Antimicrobial susceptibility testing was performed for 46.5% (47/101) of *Brucella* spp. isolates and 36.5% (23/63) of *Burkholderia pseudomallei* isolates by disk diffusion only.

In antimicrobial susceptibility testing of *Brucella* spp., the resistance rate for gentamycin was 0.0%. In the antimicrobial susceptibility testing of *Burkholderia pseudomallei*, the resistance rate for trimethoprim-sulfamethoxazole was 45.5%, and the resistance rate to ceftazidime and imipenem was 0.0% (Supplementary Table S14).

### Standardization of antimicrobial susceptibility testing

**Figure 1:**
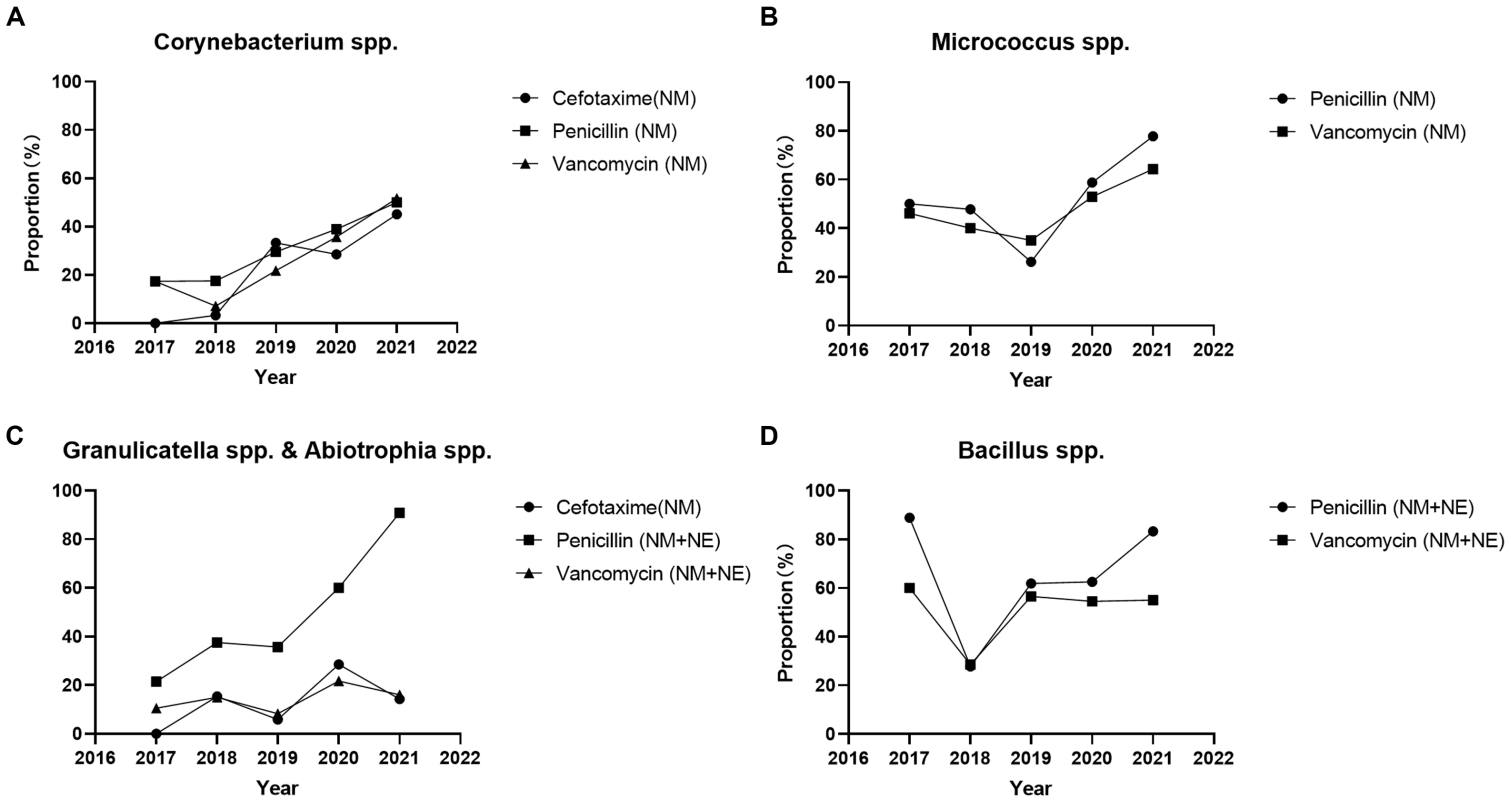
Proportion of broth microdilution method and E-test method in the antimicrobial susceptibility testing results of Infrequently Isolated or Fastidious Bacteria strains isolated from blood specimens from 2017 to 2021. Total antimicrobial susceptibility test results *n* < 10 were not included. **(A)** The proportion of broth microdilution method in antimicrobial susceptibility testing results of *Corynebacterium* spp. strains were increased significantly for cefotaxime (0.0% vs. 45.2% *p* < 0.05), penicillin (17.4% vs. 50.0% *p* < 0.05), and vancomycin (17.4% vs. 51.7% *p* < 0.001). **(B)** The proportion of broth microdilution method in antimicrobial susceptibility testing results of *Micrococcus* spp. strains were increased significantly for penicillin (50.0% vs. 77.8% *p* < 0.05), and no significant change for vancomycin (46.2% vs. 64.3% *p* = 0.142). **(C)** The proportion of broth microdilution method in antimicrobial susceptibility testing results of *Abiotrophia* spp. and *Granulicatella* spp. strains were increased significantly for penicillin (14.3% vs. 86.4% *p* < 0.001), and no significant change for cefotaxime (0.0% vs. 14.3% *p* = 0.515) and vancomycin (10.5% vs. 16.0% *p* = 0.684). **(D)** The proportion of broth microdilution method in the antimicrobial susceptibility testing results of *Bacillus* spp. strains were no significant change for penicillin (88.9% vs. 82.2% *p* = 1) and vancomycin (60.0% vs. 45.0% *p* = 1). Total antimicrobial susceptibility test results *n* < 10 were not included. NM, Microbroth dilution method; NE, E-test method.

According to CLSI M45 A3, antimicrobial susceptibility testing of *Aeromonas* spp. can be performed using disk diffusion and broth microdilution method. For other Infrequently Isolated or Fastidious Bacteria, only the broth microdilution method and is recommended. We defined not recommended by CLSI M45 A3 as non-standard method. The proportion of broth microdilution method in antimicrobial susceptibility testing results of *Corynebacterium* spp. strains increased significantly throughout the study period (2017 vs. 2021) for cefotaxime (0.0% vs. 45.2% *p* < 0.05), penicillin (17.4% vs. 50.0% *p* < 0.05), and vancomycin (17.4% vs. 51.7% *p* < 0.001). The proportion of broth microdilution method in antimicrobial susceptibility testing results of *Micrococcus* spp. strains increased significantly for penicillin (50.0% vs. 77.8% *p* < 0.05), whereas no significant change was observed for vancomycin (46.2% vs. 64.3% *p* = 0.142). The proportion of broth microdilution method in antimicrobial susceptibility testing results of *Abiotrophia* spp. and *Granulicatella* spp. strains increased significantly for penicillin (14.3% vs. 86.4% *p* < 0.001), and no significant change were observed for cefotaxime (0.0% vs. 14.3% *p* = 0.515) and vancomycin (10.5% vs. 16.0% *p* = 0.684). The proportion of broth microdilution method in the antimicrobial susceptibility testing results of *Bacillus* spp. strains showed no significant change for penicillin (88.9% vs. 82.3% *p* = 1) and vancomycin (60.0% vs. 45.0% *p* = 1) (Figure 1). Total antimicrobial susceptibility test results *n* < 10 were not included.

## Discussion

Infrequently Isolated or Fastidious Bacteria are often ignored in clinical practice due to low detection rates. However, some species of Infrequently Isolated or Fastidious Bacteria are more pathogenic than common bacteria, such as *Brucella* spp. and *Burkholderia pseudomallei*, which are also considered as “Potential Bacterial Agents for Bioterrorism.”

*Burkholderia pseudomallei* was first reported in the early 20th century and is distributed in tropical and subtropical regions. The mortality rate of sepsis caused by *Burkholderia pseudomallei* is as high as 40.0% even after antimicrobial treatment. The report by Limmathurotsakul et al. showed that diabetes is an important risk factor for *Burkholderia pseudomallei* infection. The report by Syed et al. pointed out that the prevalence of *Burkholderia pseudomallei* infection is increasing (Limmathurotsakul et al., 2016; Syed and Wooten, 2021; Brangsch et al., 2022). *Brucella* spp. is a facultative intracellular infectious pathogen that can infect major organs such as the heart and lungs, leading to infective endocarditis and various respiratory diseases, ultimately leading to heart failure. According to bio-safety regulations, experiments on living *Brucella* spp. need to be conducted in a bio-safety level three laboratory. However, in a report of occupational exposure to air-contaminated *Brucella* spp. that occurred in a bio-safety level three laboratory, the infection rate was as high as 43.6% (Koruk et al., 2012; Zhou et al., 2022). Therefore, we believe that in clinical laboratories, which are usually bio-safety level two, the isolation of highly pathogenic Infrequently Isolated or Fastidious Bacteria such as *Brucella* spp. requires more attention. And it is necessary to improve the standardization of bacterial testing to prevent occupational exposure incidents.

In this study, *Aeromonas hydrophila*, *Corynebacterium striatum*, and *Micrococcus luteus* were the top three of most isolated bacterial genera. *Aeromonas hydrophila* was once considered an environmental contaminant strain in clinical testing. However, current research shows that *Aeromonas hydrophila* often causes gastroenteritis, soft tissue infection, and can lead to sepsis when immunity is insufficient (Jones and Wilcox, 1995). In our study, the isolation of *Corynebacterium striatum* and its proportion in Infrequently Isolated or Fastidious Bacteria increased significantly from 2017 to 2021. *Corynebacterium striatum* infection is common in patients with underlying medical conditions such as hematological malignancies, solid tumors, and diabetes. Nosocomial infections caused by *Corynebacterium striatum* often occur in patients undergoing catheter intervention (Abe et al., 2021). *Micrococcus luteus* infection can cause liver and brain abscesses, bacteremia, and septic arthritis, and is common in patients with malignant tumors and immunodeficiency (Zhu et al., 2021).

*Corynebacterium striatum* was generally resistant to ciprofloxacin (95.6%) in our study, which is consistent with the result reported by Wang Y. et al. (2021) that 100.0% (410/410) of *Corynebacterium striatum* were resistant to ciprofloxacin. In our study, the resistance rate of *Micrococcus luteus* to erythromycin was 36.3%, and the resistance rate to clindamycin was 17.5%, which may indicate that a high number of infections caused by *Micrococcus luteus* resistant to ERY and CLI may fail therapy. The report by Bianco et al. in Italy showed that *Bacillus cereus* isolated from human bacteremia carries resistance genes to penicillin and trimethoprim, resulting in a 100% resistance rate to both of these antimicrobial agents (Bianco et al., 2021). We believe this might be the reason why the *Bacillus cereus* isolated in this study showed high levels of resistance to penicillin (88.0% for broth microdilution method, 100.0% for disk diffusion method) and trimethoprim-sulfamethoxazole (61.5% for disk diffusion method).

Due to the use of antimicrobial agents, the concentration of these agents in environments like water and food has increased rapidly (Jampani et al., 2024; Sun et al., 2024). This has led to a rapid increase in antimicrobial resistance levels in the environment, including among common bacteria and Infrequently Isolated or Fastidious Bacteria (Wyres and Holt, 2018; Silva-Santana et al., 2021; Algammal et al., 2022b; Denissen et al., 2022). *Corynebacterium striatum* appears to be an emerging MDR pathogen in among *Corynebacterium* spp. In the report of Yamamuro et al., *Corynebacterium striatum* and *Corynebacterium jeikeium* were less susceptible than other species to penicillin, ceftriaxone, meropenem, erythromycin, and ciprofloxacin (Yamamuro et al., 2021). In a survey in China, 96.2% of *Corynebacterium striatum* isolates were MDR strains (Wang et al., 2022). In the study by McMullen et al., the *Corynebacterium striatum* they isolated were highly resistant to antibacterial agents except vancomycin, linezolid and daptomycin. Surprisingly, all isolates became less susceptible to daptomycin and had elevated MIC50 and MIC90 to vancomycin after overnight incubation with daptomycin (McMullen et al., 2017). In the epidemiological investigation of *Aeromonas* spp., a low resistance levels were observed in our study. The studies by Nolla-Salas et al. and Sun et al. both showed that trimethoprim-sulfamethoxazole, ciprofloxacin and cefepime have good activity. However, Sun et al. found 9 MDR strains (15.5% 9/58) (Nolla-Salas et al., 2017; Sun et al., 2021). For *Micrococcus luteus*, Zhu et al. found low rates of resistance to linezolid, cefoxitin, rifampin, cefazolin and penicillin. However, resistance to gentamycin, cephalosporins, levofloxacin and carbapenems strains have also emerged. and blood stream infection (BSI) caused by *Micrococcus luteus* is increasing rapidly (Zhu et al., 2021). In short, *Corynebacterium* spp. is becoming a new MDR bacterium among Infrequently Isolated or Fastidious Bacteria. Although *Aeromonas* spp. and *Micrococcus* spp. were currently at a low resistance level, the emergence of MDR strains is still a threat worthy of attention.

Antimicrobial susceptibility testing was performed on less than 50.0% of the isolates in our study. We believe that some clinical laboratories send Potential Bacterial Agents of Bioterrorism strains to the Centers for Disease Control and Prevention for antimicrobial susceptibility testing due to insufficient bio-safety levels. However, this study did not include data from the Chinese Center for Disease Control and Prevention. *Aeromonas* spp. isolates antimicrobial susceptibility testing was performed on less than 30.0% of the isolates. *Aeromonas* spp. was once considered an environmental contaminant and in accordance with CLSI M45 A3, antimicrobial susceptibility testing is usually limited to isolates from extraintestinal sites.

Monitoring bacterial resistance levels is considered a necessary means to control bacterial resistance rate, so global and national bacterial resistance monitoring systems such as GLASS, CRASS, NARMS, and EUCAST have been established. Clinical laboratories are the important source of bacterial resistance data, so we believe that reference standards are crucial for antimicrobial susceptibility testing. Except for *Aeromonas* spp., the antimicrobial susceptibility testing method recommended by CLSI M45 A3 is the broth microdilution method. However, most hospitals in our study used the disk diffusion method for antimicrobial susceptibility testing. We attempted to analyze the difference in resistance rates between the broth microdilution method and the disk diffusion method. In our study, the resistance rate of *Corynebacterium* spp., *Abiotrophia* spp. and *Granulicatella* spp. and *Bacillus* spp. for penicillin by the broth dilution method was significantly lower than that by the disk diffusion method. There were no significant differences between the broth dilution and disk diffusion methods in the results of other antimicrobial susceptibility testing. In this study, no BSI strains of Infrequently Isolated or Fastidious Bacteria were obtained, so we were unable to verify whether the antimicrobial susceptibility testing results of the same strain by the broth microdilution method and the disk diffusion method were significantly different. Due to the small sample size and inconsistent breakpoints, we believe that this statistical result cannot prove whether there was a significant difference in antimicrobial susceptibility results between the broth microdilution method and the disk diffusion method. We will expand the sample size in future studies to evaluate the differences between broth microdilution and disk diffusion methods.

We have noticed that EUCAST Version 14.0 provides breakpoints for the disk diffusion method for most bacteria included in this study. For evaluation, we compared the breakpoints for the broth microdilution method in CLSI M45 A3 and EUCAST Version 14.0, and we found that the breakpoints specified in EUCAST Version 14.0 are lower than those in CLSI M45 A3. For example, the breakpoint of ceftazidime in *Aeromonas* spp. in CLSI is *R* ≥ 16, while in EUCAST it is *R* > 4. The same situation exists in other bacterial genera. For example, the breakpoint of ciprofloxacin in *Corynebacterium* spp. in CLSI is *R* ≥ 4, but in EUCAST it is *R* > 1. The breakpoint of rifampicin in CLSI is *R* ≥ 4, but in EUCAST it is *R* > 0.06. In view of the huge difference in breakpoints for the broth microdilution method in CLSI M45 A3 and EUCAST Version 14.0, we believe that it is inappropriate to use CLSI M45 for the broth microdilution method and EUCAST for the disk diffusion method. And since common bacteria usually use the CLSI M100 standard, we believe that it is inappropriate to use the EUCAST Version 14.0 standard for Infrequently Isolated or Fastidious Bacteria, which may lead to an abnormal increase in resistance rates. This is a challenge for bacterial resistance research and epidemiological investigations (The European Committee on Antimicrobial Susceptibility Testing, 2024).

Testing standards for common bacteria, such as CLSI M100, specify breakpoints for broth microdilution and disk diffusion methods. Clinical laboratories can choose appropriate testing methods based on economic and medical environments. The disk diffusion method is not recommended by CLSI M45 A3 as it has not been fully studied. However, some clinical laboratories do not have the ability to perform antimicrobial susceptibility testing using broth microdilution methods. We believe that the disk diffusion method is suitable for antimicrobial susceptibility testing of Infrequently Isolated or Fastidious Bacteria, but it lacks appropriate breakpoint criteria. Therefore, we hope that our research can provide a reference for the breakpoint standard of the disk diffusion method for Infrequently Isolated or Fastidious Bacteria. We look forward to CLSI launching the breakpoint of the Infrequently Isolated or Fastidious Bacteria disc diffusion method as soon as possible.

## Data availability statement

The original contributions presented in the study are included in the article/Supplementary material, further inquiries can be directed to the corresponding author.

## Ethics statement

The studies involving humans were approved by Ethics Committee of Scientific Research Project Review of the First Affiliated Hospital of Guangzhou Medical University. The studies were conducted in accordance with the local legislation and institutional requirements. The human samples used in this study were acquired from primarily isolated as part of your previous study for which ethical approval was obtained. Written informed consent for participation was not required from the participants or the participants’ legal guardians/next of kin in accordance with the national legislation and institutional requirements.

## Author contributions

NH: Writing – review & editing, Writing – original draft, Visualization, Validation, Software, Project administration, Methodology, Investigation, Formal analysis, Data curation, Conceptualization. XY: Writing – review & editing, Visualization, Validation, Supervision, Methodology, Investigation, Data curation. AH: —. JC: Writing – review & editing, Visualization, Formal analysis. YG: Writing – review & editing, Supervision. JL: Writing – review & editing, Supervision. LY: Writing – review & editing, Supervision. ChuZ: Writing – review & editing, Supervision. JW: Writing – review & editing, Supervision. YW: Writing – review & editing, Supervision. ML: Writing – review & editing, Supervision. YL: Writing – review & editing, Investigation. SX: Writing – review & editing, Supervision. ChaZ: Writing – review & editing, Supervision.
