# Supplementary material for: Practice of standardization of CLSI M45 A3 antimicrobial susceptibility testing of Infrequently Isolated or Fastidious Bacteria strains isolated from blood specimens in Guangdong Province 2017–2021
Source: Front Microbiol. 2024 Apr 29;15:1335169. doi: 10.3389/fmicb.2024.1335169 (PMC11089136; doi:10.3389/fmicb.2024.1335169)
Supplement: Supplementary file 1 [file Data_Sheet_1.ZIP › TABLE S1.pdf]

TABLE S1 Abbreviations for Antimicrobial Agents Listed

Agent Abbreviation	Antimicrobial Agents
AMC	amoxicillin/clavalanse
AMK	amikacin
AMP	ampicillin
CAZ	ceftazidime
CHL	chloramphenicol
CIP	ciprofloxacin
CLI	clindamycin
CTX	cefotaxime
DOX	doxycycline
ERY	erythromycin
GEN	gentamycin
IPM	imipenem
PEN	penicillin
RIF	rifampin
SXT	trimethoprim/sulfamethoxazole
TCY	tetracycline
VAN	vancomycin
