# Supplementary material for: Practice of standardization of CLSI M45 A3 antimicrobial susceptibility testing of Infrequently Isolated or Fastidious Bacteria strains isolated from blood specimens in Guangdong Province 2017–2021
Source: Front Microbiol. 2024 Apr 29;15:1335169. doi: 10.3389/fmicb.2024.1335169 (PMC11089136; doi:10.3389/fmicb.2024.1335169)
Supplement: Supplementary file 1 [file Data_Sheet_1.ZIP › TABLE S2.pdf]

TABLE S1 The composition of rare bacteria was isolated from blood samples

Organism	2017 (n=401)		2018 (n=415)		2019 (n=530)		2020 (n=472)		2021 (n=694)		2017~2021 (n=2512)		P-value
	**No. of strain**	**%**	**No. of strain**	**%**	**No. of strain**	**%**	**No. of strain**	**%**	**No. of strain**	**%**	**No. of strain**	**%**	
Aeromonas spp.	178	44.39%	173	41.69%	218	41.13%	173	36.65%	191	27.52%	933	37.14%	<0.001
A. hydrophila	113	28.18%	107	25.78%	140	26.42%	104	22.03%	111	15.99%	575	22.89%	<0.001
A. caviae	25	6.23%	25	6.02%	32	6.04%	28	5.93%	35	5.04%	145	5.77%	0.404
A. sobria	26	6.48%	23	5.54%	29	5.47%	26	5.51%	25	3.60%	129	5.14%	0.029
Other*	14	3.49%	18	4.34%	17	3.21%	15	3.18%	20	2.88%	84	3.34%	0.575
Corynebacteri	49	12.22	73	17.59	84	15.85	95	20.13	187	26.95	488	19.43	<0.00

um spp.		%		%		%		%		%		%	1
C. striatum	15	3.74%	28	6.75%	37	6.98%	53	11.23%	132	19.02%	265	10.55%	<0.001
C. jeikeium	2	0.50%	5	1.20%	8	1.51%	10	2.12%	14	2.02%	39	1.55%	<0.001
C. afermentans	1	0.25%	2	0.48%	1	0.19%	5	1.06%	16	2.31%	25	1.00%	0.044
Other*	31	7.73%	38	9.16%	38	7.17%	27	5.72%	25	3.60%	159	6.33%	0.003
Micrococcus spp.	53	13.22%	49	11.81%	63	11.89%	24	5.08%	55	7.93%	244	9.71%	0.005
M. luteus	45	11.22%	45	10.84%	56	10.57%	24	5.08%	48	6.92%	218	8.68%	0.014
Other*	8	2.00%	4	0.96%	7	1.32%	0	0.00%	7	1.01%	26	1.04%	0.176
Potential	22	5.49%	31	7.47%	25	4.72%	25	5.30%	65	9.37%	168	6.69%	0.022

Bacterial Agents of Bioterrorism													
Brucella spp..	16	3.99%	20	4.82%	11	2.08%	12	2.54%	42	6.05%	101	4.02%	0.142
Burkholderia pseudomallei	5	1.25%	10	2.41%	14	2.64%	12	2.54%	22	3.17%	63	2.51%	0.048
Other*	1	0.25%	1	0.24%	0	0.00%	1	0.21%	1	0.14%	4	0.16%	1
Abiotrophia spp. & Granulicatella spp..	22	5.49%	27	6.51%	41	7.74%	34	7.20%	41	5.91%	165	6.57%	0.773
G. adiacens	14	3.49%	21	5.06%	31	5.85%	25	5.30%	28	4.03%	119	4.74%	0.652
A. defectiva	6	1.50%	6	1.45%	8	1.51%	8	1.69%	10	1.44%	38	1.51%	<0.00

													1
Other*	2	0.50%	0	0.00%	2	0.38%	1	0.21%	3	0.43%	8	0.32%	0.149
Bacillus spp.	16	3.99%	22	5.30%	25	4.72%	28	5.93%	53	7.64%	144	5.73%	0.022
B. cereus	4	1.00%	13	3.13%	13	2.45%	21	4.45%	41	5.91%	92	3.66%	0.142
B. subtilis	10	2.49%	6	1.45%	6	1.13%	3	0.64%	2	0.29%	27	1.07%	0.048
Other*	2	0.50%	3	0.72%	6	1.13%	4	0.85%	10	1.44%	25	1.00%	0.694
Other*	61	15.21%	40	9.64%	74	13.96%	93	19.70%	102	14.70%	370	14.73%	0.818

***Some strains could not be identified as species, but only as genus, so they were classified as Other.**
