# Supplementary material for: Practice of standardization of CLSI M45 A3 antimicrobial susceptibility testing of Infrequently Isolated or Fastidious Bacteria strains isolated from blood specimens in Guangdong Province 2017–2021
Source: Front Microbiol. 2024 Apr 29;15:1335169. doi: 10.3389/fmicb.2024.1335169 (PMC11089136; doi:10.3389/fmicb.2024.1335169)
Supplement: Supplementary file 1 [file Data_Sheet_1.ZIP › TABLE S3.pdf]

TABLE S3 Susceptibility of *Aeromonas spp.* to antimicrobial agents

Antimicrobial agent	*Aeromonas spp.* （n=252）				*A. hydrophila* （n=153）				*A. caviae* （n=39）				*A. sobria* （n=9）			
	No. of strain	R(%)	I(%)	S(%)	No. of strain	R(%)	I(%)	S(%)	No. of strain	R(%)	I(%)	S(%)	No. of strain	R(%)	I(%)	S(%)
Cefuroxime[ND]	13	46.2	0	53.8	8	50	0	50	-	-	-	-	1	100	0	0
Cefoxitin[ND]	13	46.2	7.7	46.2	9	55.6	11.1	33.3	-	-	-	-	1	100	0	0
Cefotaxime[ND]	10	30	10	60	7	42.9	14.3	42.9	-	-	-	-	3	0	0	100
Cefotaxime[NM]	151	19.2	0.7	80.2	85	17.6	1.2	81.2	30	33.3	0	66.7	-	-	-	-
Ceftazidime[ND]	23	26.1	0	73.9	16	25	0	75	1	0	0	100	-	-	-	-
Imipenem[ND]	41	34.1	26.8	39	22	27.3	36.4	36.4	4	50	0	50	4	100	0	0
Aztreonam[ND]	19	21.1	0	78.9	12	16.7	0	83.3	1	0	0	100	-	-	-	-
Amikacin[ND]	12	8.3	0	91.7	7	0	0	100	1	0	0	100	-	-	-	-

Gentamycin[ND]	46	4.3	0	95.7	34	0	0	100	3	33.3	0	66.7	1	0	0	100
Ciprofloxacin[ND]	11	0	18.2	81.8	7	57.1	14.3	28.6	1	0	0	100	-	-	-	-
Tetracycline[ND]	4	25	0	75	2	0	0	100	-	-	-	-	-	-	-	-
Trimethoprim/ sulfamethoxazole[ND]	8	50	0	50	5	60	0	40	-	-	-	-	-	-	-	-
Chloramphenicol[ND]	6	16.7	16.7	66.7	5	20	0	80	-	-	-	-	-	-	-	-

NM: microbroth dilution method; ND: The result of disk diffusion test methods; -: not measured;
