# Supplementary material for: Practice of standardization of CLSI M45 A3 antimicrobial susceptibility testing of Infrequently Isolated or Fastidious Bacteria strains isolated from blood specimens in Guangdong Province 2017–2021
Source: Front Microbiol. 2024 Apr 29;15:1335169. doi: 10.3389/fmicb.2024.1335169 (PMC11089136; doi:10.3389/fmicb.2024.1335169)
Supplement: Supplementary file 1 [file Data_Sheet_1.ZIP › TABLE S4.pdf]

TABLE S4 Susceptibility of *Corynebacterium spp.* to antimicrobial agents

Antimicrobial agent	*Corynebacterium spp.* (n=410)				*C. striatum* (n=206)				*C. jeikeium* (n=34)				*C. afermentans* (n=18)			
	No. of strain	R(%)	I(%)	S(%)	No. of strain	R(%)	I(%)	S(%)	No. of strain	R(%)	I(%)	S(%)	No. of strain	R(%)	I(%)	S(%)
Penicillin ND	213	82.2	0	17.8	88	94.3	0	5.7	20	90	0	10	9	77.8	0	22.2
Penicillin NM	115	53	33.9	13	70	61.4	30	8.6	9	66.7	11.1	22.2	3	100	0	0
Cefotaxime ND	86	58.1	8.1	33.7	32	81.2	6.2	12.5	8	25	25	50	3	100	0	0
Cefotaxime NM	30	70	3.3	26.7	18	88.9	0	11.1	1	100	0	0	-	-	-	-
Vancomycin ND	274	0	0	100	123	0	0	100	25	0	0	100	11	0	0	100
Vancomycin NM	120	0	0	100	78	0	0	100	7	0	0	100	5	0	0	100
Gentamycin ND	165	26.7	5.5	67.9	73	27.4	9.6	63	20	35	0	65	9	33.3	11.1	55.6

Erythromycin ND	208	67.8	23.6	8.7	77	74	26	0	16	68.8	25	6.2	11	90.9	9.1	0
Ciprofloxacin ND	206	84	1.9	14.1	90	95.6	2.2	2.2	19	78.9	0	21.1	13	84.6	0	15.4
Doxycycline ND	23	4.3	0	95.7	10	10	0	90	5	0	0	100	2	0	0	100
Tetracycline ND	162	13	5.6	81.5	78	14.1	6.4	79.5	15	13.3	0	86.7	9	0	11.1	88.9
Clindamycin ND	272	86.4	7.4	6.2	121	92.6	5.8	1.7	23	82.6	8.7	8.7	12	91.7	8.3	0
Trimethoprim/ Sulfamethoxazolel ND	151	55.6	9.9	34.4	54	59.3	7.4	33.3	13	76.9	7.7	15.4	6	66.7	0	33.3
Rifampin ND	155	22.6	0.6	76.8	61	1.6	1.6	96.7	11	9.1	0	90.9	9	66.7	0	33.3

NM: microbroth dilution method; ND: disk diffusion test methods; -: not measured;
