# Supplementary material for: Practice of standardization of CLSI M45 A3 antimicrobial susceptibility testing of Infrequently Isolated or Fastidious Bacteria strains isolated from blood specimens in Guangdong Province 2017–2021
Source: Front Microbiol. 2024 Apr 29;15:1335169. doi: 10.3389/fmicb.2024.1335169 (PMC11089136; doi:10.3389/fmicb.2024.1335169)
Supplement: Supplementary file 1 [file Data_Sheet_1.ZIP › TABLE S5.pdf]

TABLE S5 Susceptibility of *Micrococcus spp.* to antimicrobial agents

Antimicrobial agent	*Micrococcus spp.* （n=210）				*M. luteus* （n=189）			
	No. of strain	R(%)	I(%)	S(%)	No. of strain	R(%)	I(%)	S(%)
Penicillin ND	77	15.6	0	84.4	65	13.8	0	86.2
Penicillin NM	72	18.1	0	81.9	64	18.8	0	81.2
Vancomycin ND	87	0	0	100	71	0	0	100
Vancomycin NM	73	0	0	100	64	0	0	100
Erythromycin ND	130	36.9	15.4	47.7	113	36.3	16.8	46.9
Clindamycin ND	120	18.3	12.5	69.2	103	17.5	14.6	68

NM: microbroth dilution method; ND: disk diffusion test methods; -: not measured;
