# Supplementary material for: Practice of standardization of CLSI M45 A3 antimicrobial susceptibility testing of Infrequently Isolated or Fastidious Bacteria strains isolated from blood specimens in Guangdong Province 2017–2021
Source: Front Microbiol. 2024 Apr 29;15:1335169. doi: 10.3389/fmicb.2024.1335169 (PMC11089136; doi:10.3389/fmicb.2024.1335169)
Supplement: Supplementary file 1 [file Data_Sheet_1.ZIP › TABLE S6.pdf]

TABLE S6 Susceptibility of *Granulicatella spp. & Abiotrophia spp.* to antimicrobial agents

Antimicrobial agent	*Granulicatella spp. & Abiotrophia spp.* （n=139）				*Granulicatella adiacens* （n=99）				*Abiotrophia sp.* （n=40）			
	**No. of strain**	**R(%)**	**I(%)**	**S(%)**	**No. of strain**	**R(%)**	**I(%)**	**S(%)**	**No. of strain**	**R(%)**	**I(%)**	**S(%)**
PenicillinND	49	44.9	0	55.1	35	45.7	0	54.3	14	42.9	0	57.1
Penicillin^{NM}	47	2.1	29.8	68.1	35	2.9	31.5	65.7	12	0	33.3	66.6
Penicillin^{NE}	3	0	0	100	1	0	0	100	2	0	0	100
AmpicillinND	42	2.4	0	97.6	26	3.8	0	96.2	16	0	0	100
CefotaximeND	52	11.5	3.8	84.6	34	14.7	2.9	82.4	18	5.6	5.6	88.9
Cefotaxime^{NM}	7	0	0	100	6	0	0	100	1	0	0	100
ImipenemND	4	0	0	100	1	0	0	100	3	0	0	100

ErythromycinND	119	58.8	10.1	31.1	83	59	10.8	30.1	36	58.3	8.3	33.3
ClindamycinND	115	51.3	11.3	37.4	80	56.2	10	33.8	35	40	14.3	45.7
CiprofloxacinND	7	14.3	14.3	71.4	1	0	100	0	6	0	0	100
ChloramphenicolND	97	3.1	0	96.9	66	4.5	0	95.5	31	0	0	100
VancomycinND	104	0	0	100	74	0	0	100	30	0	0	100
Vancomycin^{NM}	16	0	0	100	10	0	0	100	6	0	0	100
Vancomycin^{NE}	1	0	0	100	-	-	-	-	1	0	0	100

NM: microbroth dilution method; ND: disk diffusion test methods; NE: E-text; -: not measured;
