# Supplementary material for: Practice of standardization of CLSI M45 A3 antimicrobial susceptibility testing of Infrequently Isolated or Fastidious Bacteria strains isolated from blood specimens in Guangdong Province 2017–2021
Source: Front Microbiol. 2024 Apr 29;15:1335169. doi: 10.3389/fmicb.2024.1335169 (PMC11089136; doi:10.3389/fmicb.2024.1335169)
Supplement: Supplementary file 1 [file Data_Sheet_1.ZIP › TABLE S7.pdf]

TABLE S7 Susceptibility of *Bacillus spp.* to antimicrobial agents

Antimicrobial agent	*Bacillus spp.* (n=104)				*B. cereus* (n=63)				*B. subtilis* (n=25)			
	No. of strain	R(%)	I(%)	S(%)	No. of strain	R(%)	I(%)	S(%)	No. of strain	R(%)	I(%)	S(%)
$Penicillin^{ND}$	29	86.2	0	13.8	9	100	0	0	12	83.3	0	16.7
$Penicillin^{NM}$	47	38.3	31.9	29.8	36	88.9	0	11.1	6	100	0	0
$Penicillin^{NE}$	4	100	0	0	4	100	0	0	-	-	-	-
$Ampicillin^{ND}$	22	81.8	4.5	13.6	14	78.6	7.1	14.3	4	100	0	0
$Imipenem^{ND}$	28	3.6	0	96.4	14	7.1	0	92.9	11	0	0	100
$Vancomycin^{ND}$	46	0	0	100	21	0	0	100	17	0	0	100
$Vancomycin^{NM}$	44	0	0	100	34	0	0	100	3	0	0	100
$Vancomycin^{NE}$	4	0	0	100	4	0	0	100	-	-	-	-

Amikacin[ND]	20	0	0	100	11	0	0	100	5	0	0	100
Gentamycin[ND]	36	0	2.8	97.2	19	0	0	100	14	0	7.1	92.9
Erythromycin[ND]	43	14	39.5	46.5	22	9.1	45.5	45.5	16	18.8	37.5	43.8
Clindamycin[ND]	45	15.6	66.7	17.8	20	5	70	25	17	11.8	76.5	11.8
Tetracycline[ND]	26	3.8	0	96.2	12	8.3	0	91.7	9	0	0	100
Ciprofloxacin[ND]	33	15.2	51.5	33.3	20	20	55	25	9	0	11.1	88.9
Trimethoprim/sulfamethoxazole[ND]	35	71.4	5.7	22.9	13	61.5	7.7	30.8	15	80	6.7	13.3
Chloramphenicol[ND]	26	3.8	3.8	92.3	18	0	5.6	94.4	6	16.7	0	83.3
Rifampin[ND]	23	65.2	13	21.7	8	75	12.5	12.5	10	70	10	20

NM: microbroth dilution method; ND: disk diffusion test methods; NE: E-text; -: not measured;
