# Supplementary material for: Practice of standardization of CLSI M45 A3 antimicrobial susceptibility testing of Infrequently Isolated or Fastidious Bacteria strains isolated from blood specimens in Guangdong Province 2017–2021
Source: Front Microbiol. 2024 Apr 29;15:1335169. doi: 10.3389/fmicb.2024.1335169 (PMC11089136; doi:10.3389/fmicb.2024.1335169)
Supplement: Supplementary file 1 [file Data_Sheet_1.ZIP › TABLE S8.pdf]

TABLE S8 Susceptibility of Potential Bacterial Agents of Bioterrorism to antimicrobial agents

Antimicrobial agent	*Brucella spp.* (n=47)				*Antimicrobial agent*	*Burkholderia pseudomallei* (n=23)			
	No. of strain	R(%)	I(%)	S(%)		No. of strain	R(%)	I(%)	S(%)
Trimethoprim/sulfamethoxazoleND	1	0	0	100	Trimethoprim/sulfamethoxazoleND	11	45.5	18.2	36.4
GentamicinND	45	0	0	100	Amoxicillin/clavalanseND	3	100	0	0
DoxycyclineND	1	0	0	100	CeftazidimeND	15	0	0	100
StreptomycinND	-	-	-	-	ImipenemND	13	0	0	100
TetracyclineND	-	-	-	-	TetracyclineND	2	0	0	100
					DoxycyclineND	1	0	0	100

ND: The result of disk diffusion test methods; -: not measured;
