# Supplementary material for: Practice of standardization of CLSI M45 A3 antimicrobial susceptibility testing of Infrequently Isolated or Fastidious Bacteria strains isolated from blood specimens in Guangdong Province 2017–2021
Source: Front Microbiol. 2024 Apr 29;15:1335169. doi: 10.3389/fmicb.2024.1335169 (PMC11089136; doi:10.3389/fmicb.2024.1335169)
Supplement: Supplementary file 2 [file Data_Sheet_2.PDF]

TABLE S1 Interpretive criteria for inhibition zone diameter in *Aeromonas* spp.

Antimicrobial Agent	Disk Content	Diameter of Inhibition Zone (mm)			MIC (P g/mL) Interpretive Criteria		
		R	I	S	R	I	S
CXM	30ug	≤14	15-17	≥18	≤8	16	≥32
FOX	30ug	≤14	15-17	≥18	≤8	16	≥32
CTX	30ug	≤22	23-25	≥26	≤1	2	≥4
CAZ	30ug	≤17	18-20	≥21	≤4	8	≥16
IPM	10ug	≤19	20-22	≥23	≤1	2	≥4
ATM	30ug	≤17	18-20	≥21	≤4	8	≥16
AMK	30ug	≤12	13-14	≥15	≤16	32	≥64
GEN	10ug	≤12	13-14	≥15	≤4	8	≥16
CIP	5ug	≤15	16-20	≥21	≤1	2	≥4
TCY	30ug	≤11	12-14	≥15	≤4	8	≥16
SXT	1.25/23.75ug	≤10	11-15	≥16	≤2/38	-	≥4/76
CHL	30ug	≤12	13-17	≥18	≤8	16	≥32

R: Resistant, I: Intermediate, S: Sensitive. According to the National Committee for Clinical Laboratory standards. Cefuroxime (CXM); Cefoxitin (FOX); Cefotaxime (CTX); Ceftazidime (CAZ); Imipenem (IPM); Aztreonam (ATM); Amikacin (AMK); Gentamicin

(GEN); Ciprofloxacin (CIP); Tetracycline (TCY); Trimethoprim-Sulfamethoxazole (SXT); Chloramphenicol (CHL). -: not measured.

TABLE S2 Interpretive criteria for inhibition zone diameter in *Corynebacterium* spp.

**Antimicrobial Agent**	**Disk Content**	**Diameter of Inhibition Zone (mm)**			**MIC (P g/mL) Interpretive Criteria**		
		**R**	**I**	**S**	**R**	**I**	**S**
PEN	10ug	-	-	≥29	≤0.12	0.25-2	≥4
CTX	30ug	≤22	23-25	≥26	≤1	2	≥4
VAN	30ug	-	-	≥15	≤2	-	-
GEN	10ug	≤12	13-14	≥15	≤4	8	≥16
ERY	15ug	≤12	13-14	≥15	≤0.5	1	≥2
CIP	5ug	≤21	22-25	≥26	≤1	2	≥4
DOX	30ug	≤10	11-13	≥14	≤4	8	≥16
TCY	30ug	≤11	12-14	≥15	≤4	8	≥16
CLI	2ug	≤14	15-20	≥21	≤0.5	1-2	≥4
SXT	1.25/23.75ug	≤10	11-15	≥16	≤2/38	-	≥4/76
RIF	5ug	≤16	17-19	≥20	≤1	2	≥4

R: Resistant, I: Intermediate, S: Sensitive. According to the National Committee for Clinical Laboratory standards. Penicillin (PEN);

Cefotaxime (CTX); Vancomycin (VAN); Gentamicin (GEN); Erythromycin (ERY); Ciprofloxacin (CIP); Doxycycline (DOX); Tetracycline (TCY); Clindamycin (CLI);Trimethoprim-Sulfamethoxazole (SXT); Rifampin (RIF). -: not measured.

TABLE S3 Interpretive criteria for inhibition zone diameter in *Micrococcus* spp.

Antimicrobial Agent	Disk Content	Diameter of Inhibition Zone (mm)			MIC (P g/mL) Interpretive Criteria		
		R	I	S	R	I	S
PEN	10ug	-	-	≥29	≤0.12	-	≥0.25
VAN	30ug	-	-	≥15	≤2	-	-
ERY	15ug	≤13	14-22	≥23	≤0.5	1-4	≥8
CLI	5ug	≤14	15-20	≥21	≤0.5	1-2	≥4

R: Resistant, I: Intermediate, S: Sensitive. According to the National Committee for Clinical Laboratory standards. Penicillin (PEN); Vancomycin (VAN); Erythromycin(ERY); Clindamycin (CLI). -: not measured.

TABLE S4 Interpretive criteria for inhibition zone diameter in *Granulicatella* spp. & *Abiotrophia* spp..

Antimicrobial Agent	Disk Content	Diameter of Inhibition Zone (mm)			MIC (ug/mL) Interpretive Criteria			MIC (ug/mL) of E-tset Interpretive Criteria		
		R	I	S	R	I	S	R	I	S
PEN	10ug	-	-	≥29	≤0.12	0.25-2	≥4	≤0.12	-	≥0.25
AMP	10ug	≤13	14-16	≥17	≤0.25	0.5-4	≥8	-	-	-
CTX	30ug	≤22	23-25	≥26	≤1	2	≥4	-	-	-
IPM	10ug	≤19	20-22	≥23	≤0.5	1	≥2	-	-	-
ERY	15ug	≤13	14-22	≥23	≤0.25	0.5	≥1	-	-	-
CLI	2ug	≤14	15-20	≥21	≤0.25	0.5	≥1	-	-	-
CIP	5ug	≤21	22-25	≥26	≤1	2	≥4	-	-	-
CHL	30ug	≤12	13-17	≥18	≤4	-	≥8	-	-	-
VAN	30ug	-	-	≥15	≤1	-	-	≤4	-	≥32

R: Resistant, I: Intermediate, S: Sensitive. According to the National Committee for Clinical Laboratory standards. Penicillin (PEN); Ampicillin (AMP); Cefotaxime (CTX); Ciprofloxacin (IPM); Erythromycin (ERY); Clindamycin (CLI); Ciprofloxacin (CIP); Chloramphenicol (CHL); Vancomycin (VAN). -: not measured.

TABLE S5 Interpretive criteria for inhibition zone diameter in *Bacillus* spp.

Antimicrobial Agent	Disk Content	Diameter of Inhibition Zone (mm)			MIC (P g/mL) Interpretive Criteria			MIC (ug/mL) of E-tset Interpretive Criteria		
		R	I	S	R	I	S	R	I	S
PEN	10ug	-	-	≥29	≤0.12	-	≥0.25	≤0.12	-	≥0.25
AMP	10ug	≤13	14-16	≥17	≤0.25	-	≥0.5	-	-	-
IPM	10ug	≤19	20-22	≥23	≤4	8	≥16	-	-	-
VAN	30ug	-	-	≥15	≤4	-	-	≤4	-	≥32
AMK	30ug	≤14	15-16	≥17	≤16	32	≥64	-	-	-
GEN	10ug	≤12	13-14	≥15	≤4	8	≥16	-	-	-
ERY	15ug	≤13	14-22	≥23	≤0.5	1-4	≥8	-	-	-
CLI	2ug	≤14	15-20	≥21	≤0.5	1-2	≥4	-	-	-
TCY	30ug	≤11	12-14	≥15	≤4	8	≥16	-	-	-
CIP	5ug	≤21	22-25	≥26	≤1	2	≥4	-	-	-
SXT	1.25/23.75ug	≤10	11-15	≥16	≤0.5	1	≥4	-	-	-
CHL	30ug	≤12	13-17	≥18	≤8	16	≥32	-	-	-
RIF	5ug	≤16	17-19	≥20	≤1	2	≥4	-	-	-

R: Resistant, I: Intermediate, S: Sensitive. According to the National Committee for Clinical Laboratory standards. Penicillin (PEN); Ampicillin (AMP); Imipenem (IPM); Vancomycin (VAN); Amikacin (AMK); Gentamicin (GEN); Erythromycin (ERY); Clindamycin (CLI); Tetracycline (TCY); Ciprofloxacin (CIP); Trimethoprim-Sulfamethoxazole (SXT); Chloramphenicol (CHL); Rifampin (RIF). -: not measured.

TABLE S6 Interpretive criteria for inhibition zone diameter in *Brucella* spp.

Antimicrobial Agent	Disk Content	Diameter of Inhibition Zone (mm)		
		R	I	S
SXT	1.25/23.75ug	≤10	11-15	≥16
GEN	10ug	≤12	13-14	≥15
DOX	30ug	≤10	11-13	≥14

R: Resistant, I: Intermediate, S: Sensitive. According to the National Committee for Clinical Laboratory standards.

Trimethoprim-Sulfamethoxazole (SXT); Gentamicin (GEN); Doxycycline (DOX). -: not measured.

TABLE S7 Interpretive criteria for inhibition zone diameter in *Burkholderia pseudomallei*

Antimicrobial Agent	Disk Content	Diameter of Inhibition Zone (mm)		
		R	I	S
SXT	1.25/23.75ug	≤10	11-15	≥16
AMC	20ug	≤13	14-17	≥18
CAZ	30ug	≤19	18-20	≥21
IPM	10ug	≤19	20-22	≥23
TCY	30ug	≤11	12-14	≥15
DOX	30ug	≤10	11-13	≥14

R: Resistant, I: Intermediate, S: Sensitive. According to the National Committee for Clinical Laboratory standards.

Trimethoprim-Sulfamethoxazole (SXT); Amoxicillin and Clavulanate (AMC); Ceftazidime (CAZ); Imipenem (IPM); Tetracycline (TCY); Doxycycline (DOX). -: not measured.

TABLE S8 The composition of Infrequently Isolated or Fastidious Bacteria was isolated from blood samples

Organism	2017 (n=401)		2018 (n=415)		2019 (n=530)		2020 (n=472)		2021 (n=694)		2017~2021 (n=2512)		P-value
	No. of strain	%	No. of strain	%	No. of strain	%	No. of strain	%	No. of strain	%	No. of strain	%	
Aeromonas spp.	178	44.39%	173	41.69%	218	41.13%	173	36.65%	191	27.52%	933	37.14%	<0.001
A. hydrophila	113	28.18%	107	25.78%	140	26.42%	104	22.03%	111	15.99%	575	22.89%	<0.001
A. caviae	25	6.23%	25	6.02%	32	6.04%	28	5.93%	35	5.04%	145	5.77%	0.404
A. sobria	26	6.48%	23	5.54%	29	5.47%	26	5.51%	25	3.60%	129	5.14%	0.029
Other*	14	3.49%	18	4.34%	17	3.21%	15	3.18%	20	2.88%	84	3.34%	0.575
Corynebacteri	49	12.22	73	17.59	84	15.85	95	20.13	187	26.95	488	19.43	<0.00

um spp.		%		%		%		%		%		%	1
C. striatum	15	3.74%	28	6.75%	37	6.98%	53	11.23%	132	19.02%	265	10.55%	<0.001
C. jeikeium	2	0.50%	5	1.20%	8	1.51%	10	2.12%	14	2.02%	39	1.55%	<0.001
C. afermentans	1	0.25%	2	0.48%	1	0.19%	5	1.06%	16	2.31%	25	1.00%	0.044
Other*	31	7.73%	38	9.16%	38	7.17%	27	5.72%	25	3.60%	159	6.33%	0.003
Micrococcus spp.	53	13.22%	49	11.81%	63	11.89%	24	5.08%	55	7.93%	244	9.71%	0.005
M. luteus	45	11.22%	45	10.84%	56	10.57%	24	5.08%	48	6.92%	218	8.68%	0.014
Other*	8	2.00%	4	0.96%	7	1.32%	0	0.00%	7	1.01%	26	1.04%	0.176
Potential	22	5.49%	31	7.47%	25	4.72%	25	5.30%	65	9.37%	168	6.69%	0.022

Bacterial Agents of Bioterrorism													
Brucella spp.	16	3.99%	20	4.82%	11	2.08%	12	2.54%	42	6.05%	101	4.02%	0.142
Burkholderia pseudomallei	5	1.25%	10	2.41%	14	2.64%	12	2.54%	22	3.17%	63	2.51%	0.048
Other*	1	0.25%	1	0.24%	0	0.00%	1	0.21%	1	0.14%	4	0.16%	1
Abiotrophia spp. & *Granulicatella* spp.	22	5.49%	27	6.51%	41	7.74%	34	7.20%	41	5.91%	165	6.57%	0.773
G. adiacens	14	3.49%	21	5.06%	31	5.85%	25	5.30%	28	4.03%	119	4.74%	0.652
A. defectiva	6	1.50%	6	1.45%	8	1.51%	8	1.69%	10	1.44%	38	1.51%	<0.00

													1
Other*	2	0.50%	0	0.00%	2	0.38%	1	0.21%	3	0.43%	8	0.32%	0.149
Bacillus spp.	16	3.99%	22	5.30%	25	4.72%	28	5.93%	53	7.64%	144	5.73%	0.022
B. cereus	4	1.00%	13	3.13%	13	2.45%	21	4.45%	41	5.91%	92	3.66%	0.142
B. subtilis	10	2.49%	6	1.45%	6	1.13%	3	0.64%	2	0.29%	27	1.07%	0.048
Other*	2	0.50%	3	0.72%	6	1.13%	4	0.85%	10	1.44%	25	1.00%	0.694
Other*	61	15.21 %	40	9.64%	74	13.96 %	93	19.70 %	102	14.70 %	370	14.73 %	0.818

*Some strains could not be identified as species, but only as genus, so they were classified as Other.

TABLE S9 Susceptibility of *Aeromonas* spp. to antimicrobial agents

Antimicrobial agent	*Aeromonas* spp. （n=252）				*A. hydrophila* （n=153）				*A. caviae* （n=39）				*A. sobria* （n=9）			
	**No. of strain**	**R(%)**	**I(%)**	**S(%)**	**No. of strain**	**R(%)**	**I(%)**	**S(%)**	**No. of strain**	**R(%)**	**I(%)**	**S(%)**	**No. of strain**	**R(%)**	**I(%)**	**S(%)**
CXM[ND]	13	46.2	0	53.8	8	50	0	50	-	-	-	-	1	100	0	0
FOX[ND]	13	46.2	7.7	46.2	9	55.6	11.1	33.3	-	-	-	-	1	100	0	0
CTX[ND]	10	30	10	60	7	42.9	14.3	42.9	-	-	-	-	3	0	0	100
CTX[NM]	151	19.2	0.7	80.2	85	17.6	1.2	81.2	30	33.3	0	66.7	-	-	-	-
CAZ[ND]	23	26.1	0	73.9	16	25	0	75	1	0	0	100	-	-	-	-
IPM[ND]	41	34.1	26.8	39	22	27.3	36.4	36.4	4	50	0	50	4	100	0	0
ATM[ND]	19	21.1	0	78.9	12	16.7	0	83.3	1	0	0	100	-	-	-	-
AMK[ND]	12	8.3	0	91.7	7	0	0	100	1	0	0	100	-	-	-	-

GEN^ND	46	4.3	0	95.7	34	0	0	100	3	33.3	0	66.7	1	0	0	100
CIP^ND	11	0	18.2	81.8	7	57.1	14.3	28.6	1	0	0	100	-	-	-	-
TCY^ND	4	25	0	75	2	0	0	100	-	-	-	-	-	-	-	-
SXT^ND	8	50	0	50	5	60	0	40	-	-	-	-	-	-	-	-
CHL^ND	6	16.7	16.7	66.7	5	20	0	80	-	-	-	-	-	-	-	-

NM: microbroth dilution method; ND: The result of disk diffusion test methods; -: not measured;

TABLE S10 Susceptibility of *Corynebacterium* spp. to antimicrobial agents

Antimicrobial agent	***Corynebacterium* spp. （n=410）**				***C. striatum* （n=206）**				***C. jeikeium* （n=34）**				***C. afermentans* （n=18）**			
	**No. of strain**	**R(%)**	**I(%)**	**S(%)**	**No. of strain**	**R(%)**	**I(%)**	**S(%)**	**No. of strain**	**R(%)**	**I(%)**	**S(%)**	**No. of strain**	**R(%)**	**I(%)**	**S(%)**
PEN ND	213	82.2	0	17.8	88	94.3	0	5.7	20	90	0	10	9	77.8	0	22.2
PEN ^{NM}	115	53	33.9	13	70	61.4	30	8.6	9	66.7	11.1	22.2	3	100	0	0
CTX ND	86	58.1	8.1	33.7	32	81.2	6.2	12.5	8	25	25	50	3	100	0	0
CTX ^{NM}	30	70	3.3	26.7	18	88.9	0	11.1	1	100	0	0	-	-	-	-
VAN ND	274	0	0	100	123	0	0	100	25	0	0	100	11	0	0	100
VAN ^{NM}	120	0	0	100	78	0	0	100	7	0	0	100	5	0	0	100
GEN ND	165	26.7	5.5	67.9	73	27.4	9.6	63	20	35	0	65	9	33.3	11.1	55.6
ERY ND	208	67.8	23.6	8.7	77	74	26	0	16	68.8	25	6.2	11	90.9	9.1	0

CIP [ND]	206	84	1.9	14.1	90	95.6	2.2	2.2	19	78.9	0	21.1	13	84.6	0	15.4
DOX [ND]	23	4.3	0	95.7	10	10	0	90	5	0	0	100	2	0	0	100
TCY [ND]	162	13	5.6	81.5	78	14.1	6.4	79.5	15	13.3	0	86.7	9	0	11.1	88.9
CLI [ND]	272	86.4	7.4	6.2	121	92.6	5.8	1.7	23	82.6	8.7	8.7	12	91.7	8.3	0
SXT [ND]	151	55.6	9.9	34.4	54	59.3	7.4	33.3	13	76.9	7.7	15.4	6	66.7	0	33.3
RIF [ND]	155	22.6	0.6	76.8	61	1.6	1.6	96.7	11	9.1	0	90.9	9	66.7	0	33.3

NM: microbroth dilution method; ND: disk diffusion test methods; -: not measured;

TABLE S11 Susceptibility of *Micrococcus* spp. to antimicrobial agents

Antimicrobial agent	*Micrococcus* spp. （n=210）				*M. luteus* （n=189）			
	No. of strain	R(%)	I(%)	S(%)	No. of strain	R(%)	I(%)	S(%)
PEN ND	77	15.6	0	84.4	65	13.8	0	86.2
PEN NM	72	18.1	0	81.9	64	18.8	0	81.2
VAN ND	87	0	0	100	71	0	0	100
VAN NM	73	0	0	100	64	0	0	100
ERY ND	130	36.9	15.4	47.7	113	36.3	16.8	46.9
CLI ND	120	18.3	12.5	69.2	103	17.5	14.6	68

NM: microbroth dilution method; ND: disk diffusion test methods; -: not measured;

TABLE S12 Susceptibility of *Granulicatella* spp. & *Abiotrophia* spp. to antimicrobial agents

Antimicrobial agent	*Granulicatella* spp. & *Abiotrophia* spp. （n=139）				*Granulicatella adiacens* （n=99）				*Abiotrophia* spp. （n=40）			
	No. of strain	R(%)	I(%)	S(%)	No. of strain	R(%)	I(%)	S(%)	No. of strain	R(%)	I(%)	S(%)
PENND	49	44.9	0	55.1	35	45.7	0	54.3	14	42.9	0	57.1
PEN^{NM}	47	2.1	29.8	68.1	35	2.9	31.5	65.7	12	0	33.3	66.6
PEN^{NE}	3	0	0	100	1	0	0	100	2	0	0	100
AMPND	42	2.4	0	97.6	26	3.8	0	96.2	16	0	0	100
CTXND	52	11.5	3.8	84.6	34	14.7	2.9	82.4	18	5.6	5.6	88.9
CTX^{NM}	7	0	0	100	6	0	0	100	1	0	0	100
IPMND	4	0	0	100	1	0	0	100	3	0	0	100

ERYND	119	58.8	10.1	31.1	83	59	10.8	30.1	36	58.3	8.3	33.3
CLIND	115	51.3	11.3	37.4	80	56.2	10	33.8	35	40	14.3	45.7
CIPND	7	14.3	14.3	71.4	1	0	100	0	6	0	0	100
CHLND	97	3.1	0	96.9	66	4.5	0	95.5	31	0	0	100
VANND	104	0	0	100	74	0	0	100	30	0	0	100
VAN^{NM}	16	0	0	100	10	0	0	100	6	0	0	100
VAN^{NE}	1	0	0	100	-	-	-	-	1	0	0	100

NM: microbroth dilution method; ND: disk diffusion test methods; NE: E-text; -: not measured;

TABLE S13 Susceptibility of *Bacillus* spp.to antimicrobial agents

Antimicrobial agent	*Bacillus* spp. （n=104）				*B. cereus* （n=63）				*B. subtilis* （n=25）			
	No. of strain	R(%)	I(%)	S(%)	No. of strain	R(%)	I(%)	S(%)	No. of strain	R(%)	I(%)	S(%)
PEN[ND]	29	86.2	0	13.8	9	100	0	0	12	83.3	0	16.7
PEN[NM]	47	38.3	31.9	29.8	36	88.9	0	11.1	6	100	0	0
PEN[NE]	4	100	0	0	4	100	0	0	-	-	-	-
AMP[ND]	22	81.8	4.5	13.6	14	78.6	7.1	14.3	4	100	0	0
IPM[ND]	28	3.6	0	96.4	14	7.1	0	92.9	11	0	0	100
VAN[ND]	46	0	0	100	21	0	0	100	17	0	0	100
VAN[NM]	44	0	0	100	34	0	0	100	3	0	0	100
VAN[NE]	4	0	0	100	4	0	0	100	-	-	-	-

AMK[ND]	20	0	0	100	11	0	0	100	5	0	0	100
GEN[ND]	36	0	2.8	97.2	19	0	0	100	14	0	7.1	92.9
ERY[ND]	43	14	39.5	46.5	22	9.1	45.5	45.5	16	18.8	37.5	43.8
CLI[ND]	45	15.6	66.7	17.8	20	5	70	25	17	11.8	76.5	11.8
TCY[ND]	26	3.8	0	96.2	12	8.3	0	91.7	9	0	0	100
CIP[ND]	33	15.2	51.5	33.3	20	20	55	25	9	0	11.1	88.9
SXT[ND]	35	71.4	5.7	22.9	13	61.5	7.7	30.8	15	80	6.7	13.3
CHL[ND]	26	3.8	3.8	92.3	18	0	5.6	94.4	6	16.7	0	83.3
RIF[ND]	23	65.2	13	21.7	8	75	12.5	12.5	10	70	10	20

NM: microbroth dilution method; ND: disk diffusion test methods; NE: E-text; -: not measured;

TABLE S14 Susceptibility of Potential Bacterial Agents of Bioterrorism to antimicrobial agents

Antimicrobial agent	*Brucella* spp. (n=47)				Antimicrobial agent	*Burkholderia pseudomallei* (n=23)			
	No. of strain	R(%)	I(%)	S(%)		No. of strain	R(%)	I(%)	S(%)
SXT[ND]	1	0	0	100	SXT[ND]	11	45.5	18.2	36.4
GEN[ND]	45	0	0	100	AMC[ND]	3	100	0	0
DOX[ND]	1	0	0	100	CAZ[ND]	15	0	0	100
-	-	-	-	-	IPM[ND]	13	0	0	100
-	-	-	-	-	TCY[ND]	2	0	0	100
-	-	-	-	-	DOX[ND]	1	0	0	100

ND: the result of disk diffusion test methods; -: not measured;
