# Supplementary material for: Practice of standardization of CLSI M45 A3 antimicrobial susceptibility testing of Infrequently Isolated or Fastidious Bacteria strains isolated from blood specimens in Guangdong Province 2017–2021
Source: Front Microbiol. 2024 Apr 29;15:1335169. doi: 10.3389/fmicb.2024.1335169 (PMC11089136; doi:10.3389/fmicb.2024.1335169)
Supplement: Supplementary file 4 [file Data_Sheet_4.PDF]

A

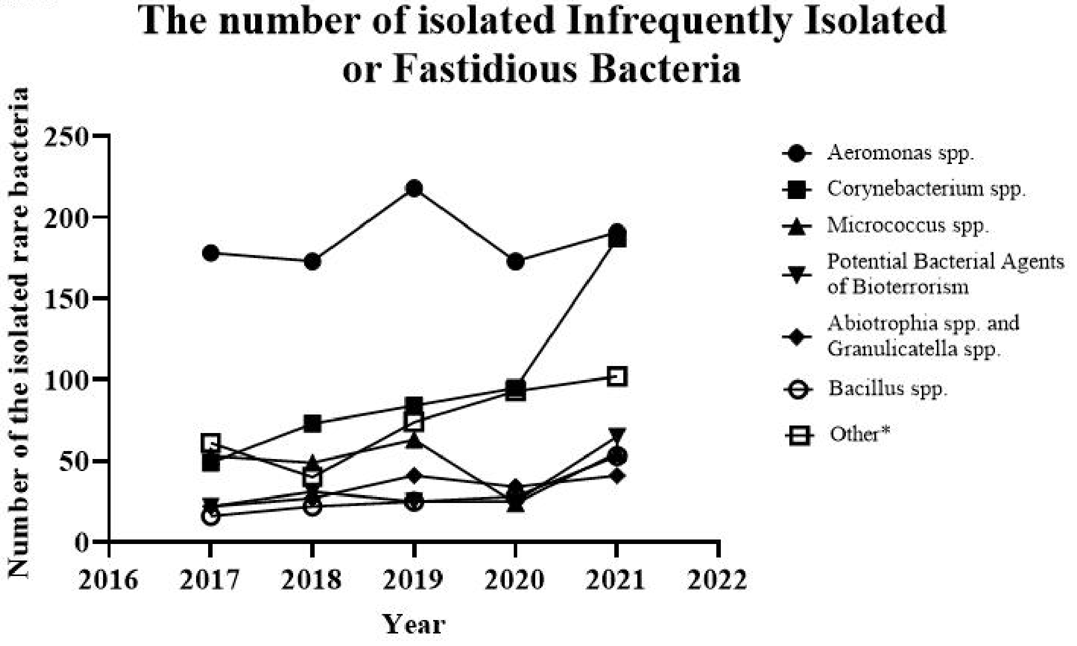

B

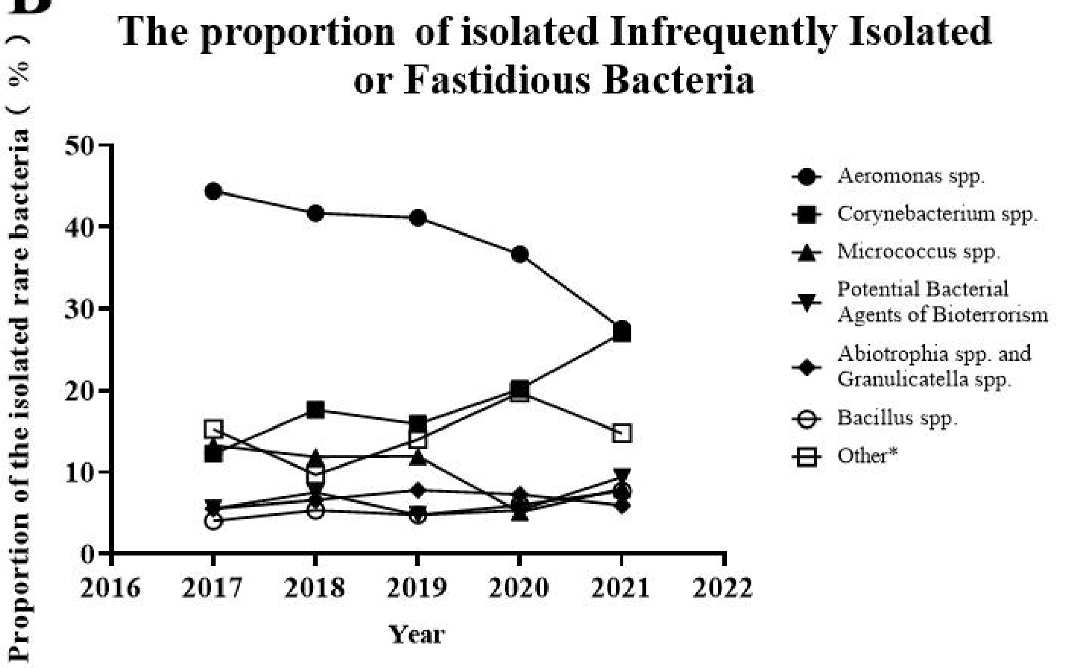

Figure S1. The number and proportion of isolated Infrequently Isolated or Fastidious Bacteria 2017 - 2021. (A) The number of isolated Infrequently Isolated or Fastidious Bacteria 2017 - 2021. The number of isolated of *Aeromonas* spp.

rose in 2019, then quickly fell back, none of the changes were significant. The number of *Corynebacterium* spp. isolated continued to rise from 2017 to 2020, and climbed rapidly significantly in 2021（ $p < 0.01$ ）. The number of isolated of *Micrococcus* spp. declined in 2020 and then recovered in 2021, none of the changes were significant. The number of isolates of *Abiotrophia* spp. and *Granulicatella* spp., *Bacillus* spp. and Potential Bacterial Agents of Bioterrorism did not change significantly, with a slight increase in 2021, none of the changes were significant. **(B)** The proportion of isolated Infrequently Isolated or Fastidious Bacteria 2017 – 2021. The proportion of Aeromonas spp. isolated continued to decrease significantly ($p<0.001$). In contrast, the proportion of Corynebacterium spp. isolated continued to significantly increase ($p<0.001$). The proportion of Micrococcus spp. isolated continued to decrease until 2020 and then increased ($p=0.05$). The proportion of isolation of Potential Bacterial Agents of Bioterrorism and Bacillus spp. increased significantly ($P<0.05$). All other Infrequently Isolated or Fastidious Bacteria had no significant changes in their isolation proportions.

* Other means other Infrequently Isolated or Fastidious Bacteria.
