# Supplementary material for: Practice of standardization of CLSI M45 A3 antimicrobial susceptibility testing of Infrequently Isolated or Fastidious Bacteria strains isolated from blood specimens in Guangdong Province 2017–2021
Source: Front Microbiol. 2024 Apr 29;15:1335169. doi: 10.3389/fmicb.2024.1335169 (PMC11089136; doi:10.3389/fmicb.2024.1335169)
Supplement: Supplementary file 5 [file Data_Sheet_5.PDF]

TABLE S1 Interpretive criteria for inhibition zone diameter in *Aeromonas* spp.

Antimicrobial Agent	Disk Content	Diameter of Inhibition Zone (mm)			MIC (P g/mL) Interpretive Criteria		
		R	I	S	S	I	R
CXM	30ug	≤14	15-17	≥18	≤8	16	≥32
FOX	30ug	≤14	15-17	≥18	≤8	16	≥32
CTX	30ug	≤22	23-25	≥26	≤1	2	≥4
CAZ	30ug	≤17	18-20	≥21	≤4	8	≥16
IPM	10ug	≤19	20-22	≥23	≤1	2	≥4
ATM	30ug	≤17	18-20	≥21	≤4	8	≥16
AMK	30ug	≤14	15-16	≥17	≤16	32	≥64
GEN	10ug	≤12	13-14	≥15	≤4	8	≥16
CIP	5ug	≤15	16-20	≥21	≤1	2	≥4
TCY	30ug	≤11	12-14	≥15	≤4	8	≥16
SXT	1.25/23.75ug	≤10	11-15	≥16	≤2/38	-	≥4/76
CHL	30ug	≤12	13-17	≥18	≤8	16	≥32

The MIC (μg/mL) of the broth microdilution method used the CLSI M45 A3. R: Resistant, I: Intermediate, S: Sensitive. According to the National Committee for Clinical Laboratory standards. Cefuroxime (CXM); Cefoxitin (FOX); Cefotaxime (CTX); Ceftazidime

(CAZ); Imipenem (IPM); Aztreonam (ATM); Amikacin (AMK); Gentamicin (GEN); Ciprofloxacin (CIP); Tetracycline (TCY); Trimethoprim-Sulfamethoxazole (SXT); Chloramphenicol (CHL). -: not measured.

TABLE S2 Interpretive criteria for inhibition zone diameter in *Corynebacterium* spp.

Antimicrobial Agent	Disk Content	Diameter of Inhibition Zone (mm)			MIC (P g/mL) Interpretive Criteria		
		R	I	S	S	I	R
PEN	10ug	-	-	≥29	≤0.12	0.25-2	≥4
CTX	30ug	≤22	23-25	≥26	≤1	2	≥4
VAN	30ug	-	-	≥15	≤2	-	-
GEN	10ug	≤12	13-14	≥15	≤4	8	≥16
ERY	15ug	≤12	13-14	≥15	≤0.5	1	≥2
CIP	5ug	≤21	22-25	≥26	≤1	2	≥4
DOX	30ug	≤10	11-13	≥14	≤4	8	≥16
TCY	30ug	≤11	12-14	≥15	≤4	8	≥16
CLI	2ug	≤14	15-20	≥21	≤0.5	1-2	≥4
SXT	1.25/23.75ug	≤10	11-15	≥16	≤2/38	-	≥4/76
RIF	5ug	≤16	17-19	≥20	≤1	2	≥4

The MIC (µg/mL) of the broth microdilution method used the CLSI M45 A3. The Diameter of Inhibition Zone (mm) of the disk diffusion method used the CLSI M100-ED32. R: Resistant, I: Intermediate, S: Sensitive. According to the National Committee for Clinical Laboratory standards. Penicillin (PEN); Cefotaxime (CTX); Vancomycin (VAN); Gentamicin (GEN); Erythromycin (ERY); Ciprofloxacin (CIP); Doxycycline (DOX); Tetracycline (TCY); Clindamycin (CLI);Trimethoprim-Sulfamethoxazole (SXT); Rifampin (RIF). -: not measured.

TABLE S3 Interpretive criteria for inhibition zone diameter in *Micrococcus* spp.

Antimicrobial Agent	Disk Content	Diameter of Inhibition Zone (mm)			MIC (P g/mL) Interpretive Criteria		
		R	I	S	S	I	R
PEN	10ug	-	-	≥29	≤0.12	-	≥0.25
VAN	30ug	-	-	≥15	≤2	-	-
ERY	15ug	≤13	14-22	≥23	≤0.5	1-4	≥8
CLI	5ug	≤14	15-20	≥21	≤0.5	1-2	≥4

The MIC (μg/mL) of the broth microdilution method used the CLSI M45 A3. The Diameter of Inhibition Zone (mm) of the disk diffusion method used the CLSI M100-ED32. R: Resistant, I: Intermediate, S: Sensitive. According to the National Committee for Clinical Laboratory standards. Penicillin (PEN); Vancomycin (VAN); Erythromycin(ERY); Clindamycin (CLI). -: not measured.

TABLE S4 Interpretive criteria for inhibition zone diameter in *Granulicatella* spp. and *Abiotrophia* spp..

Antimicrobial Agent	Disk Content	Diameter of Inhibition Zone (mm)			MIC (ug/mL) Interpretive Criteria			MIC (ug/mL) of E-tset Interpretive Criteria		
		R	I	S	S	I	R	S	I	R
PEN	10ug	-	-	≥29	≤0.12	0.25-2	≥4	≤0.12	-	≥0.25
AMP	10ug	≤13	14-16	≥17	≤0.25	0.5-4	≥8	-	-	-
CTX	30ug	≤22	23-25	≥26	≤1	2	≥4	-	-	-
IPM	10ug	≤19	20-22	≥23	≤0.5	1	≥2	-	-	-
ERY	15ug	≤13	14-22	≥23	≤0.25	0.5	≥1	-	-	-
CLI	2ug	≤14	15-20	≥21	≤0.25	0.5	≥1	-	-	-
CIP	5ug	≤21	22-25	≥26	≤1	2	≥4	-	-	-
CHL	30ug	≤12	13-17	≥18	≤4	-	≥8	-	-	-
VAN	30ug	-	-	≥15	≤1	-	-	≤4	-	≥32

The MIC (μg/mL) of the broth microdilution method used the CLSI M45 A3. The Diameter of Inhibition Zone (mm) of the disk diffusion method used the CLSI M100-ED32. R: Resistant, I: Intermediate, S: Sensitive. According to the National Committee for Clinical Laboratory standards. Penicillin (PEN); Ampicillin (AMP); Cefotaxime (CTX); Ciprofloxacin (IPM); Erythromycin (ERY); Clindamycin (CLI); Ciprofloxacin (CIP); Chloramphenicol (CHL); Vancomycin (VAN). -: not measured.

TABLE S5 Interpretive criteria for inhibition zone diameter in *Bacillus* spp.

Antimicrobial Agent	Disk Content	Diameter of Inhibition Zone (mm)			MIC (P g/mL) Interpretive Criteria			MIC (ug/mL) of E-tset Interpretive Criteria		
		R	I	S	S	I	R	S	I	R
PEN	10ug	-	-	≥29	≤0.12	-	≥0.25	≤0.12	-	≥0.25
AMP	10ug	≤13	14-16	≥17	≤0.25	-	≥0.5	-	-	-
IPM	10ug	≤19	20-22	≥23	≤4	8	≥16	-	-	-
VAN	30ug	-	-	≥15	≤4	-	-	≤4	-	≥32
AMK	30ug	≤14	15-16	≥17	≤16	32	≥64	-	-	-
GEN	10ug	≤12	13-14	≥15	≤4	8	≥16	-	-	-
ERY	15ug	≤13	14-22	≥23	≤0.5	1-4	≥8	-	-	-
CLI	2ug	≤14	15-20	≥21	≤0.5	1-2	≥4	-	-	-
TCY	30ug	≤11	12-14	≥15	≤4	8	≥16	-	-	-
CIP	5ug	≤21	22-25	≥26	≤1	2	≥4	-	-	-
SXT	1.25/23.75ug	≤10	11-15	≥16	≤0.5	1	≥4	-	-	-
CHL	30ug	≤12	13-17	≥18	≤8	16	≥32	-	-	-
RIF	5ug	≤16	17-19	≥20	≤1	2	≥4	-	-	-

The MIC (μg/mL) of the broth microdilution method used the CLSI M45 A3. The Diameter of Inhibition Zone (mm) of the disk diffusion method used the CLSI M100-ED32. R: Resistant, I: Intermediate, S: Sensitive. According to the National Committee for Clinical Laboratory standards. Penicillin (PEN); Ampicillin (AMP); Imipenem (IPM); Vancomycin (VAN); Amikacin (AMK); Gentamicin (GEN); Erythromycin (ERY); Clindamycin (CLI); Tetracycline (TCY); Ciprofloxacin (CIP); Trimethoprim-Sulfamethoxazole (SXT); Chloramphenicol (CHL); Rifampin (RIF). -: not measured.

TABLE S6 Interpretive criteria for inhibition zone diameter in *Brucella* spp.

Antimicrobial Agent	Disk Content	Diameter of Inhibition Zone (mm)		
		R	I	S
SXT	1.25/23.75ug	≤10	11-15	≥16
GEN	10ug	≤12	13-14	≥15
DOX	30ug	≤10	11-13	≥14

The MIC (μg/mL) of the broth microdilution method used the CLSI M45 A3. The Diameter of Inhibition Zone (mm) of the disk diffusion method used the CLSI M100-ED32. R: Resistant, I: Intermediate, S: Sensitive. According to the National Committee for Clinical Laboratory standards. Trimethoprim-Sulfamethoxazole (SXT); Gentamicin (GEN); Doxycycline (DOX). -: not measured.

TABLE S7 Interpretive criteria for inhibition zone diameter in *Burkholderia pseudomallei*

Antimicrobial Agent	Disk Content	Diameter of Inhibition Zone (mm)		
		R	I	S
SXT	1.25/23.75ug	≤10	11-15	≥16
AMC	20ug	≤13	14-17	≥18
CAZ	30ug	≤19	18-20	≥21
IPM	10ug	≤19	20-22	≥23
TCY	30ug	≤11	12-14	≥15
DOX	30ug	≤10	11-13	≥14

The MIC (μg/mL) of the broth microdilution method used the CLSI M45 A3. The Diameter of Inhibition Zone (mm) of the disk diffusion method used the CLSI M100-ED32. R: Resistant, I: Intermediate, S: Sensitive. According to the National Committee for Clinical Laboratory standards. Trimethoprim-Sulfamethoxazole (SXT); Amoxicillin and Clavulanate (AMC); Ceftazidime (CAZ); Imipenem (IPM); Tetracycline (TCY); Doxycycline (DOX). -: not measured.

TABLE S8 Infrequently isolated or fastidious bacteria isolated from blood samples in Guangdong Province between 2017 and 2021

Organism	2017 (n=401)		2018 (n=415)		2019 (n=530)		2020 (n=472)		2021 (n=694)		2017~2021 (n=2512)		P-value
	No. of strain	%	No. of strain	%	No. of strain	%	No. of strain	%	No. of strain	%	No. of strain	%	
Aeromonas spp.	178	44.4%	173	41.7%	218	41.1%	173	36.7%	191	27.5%	933	37.1%	<0.001
A. hydrophila	113	28.2%	107	25.8%	140	26.4%	104	22.0%	111	16.0%	575	22.9%	<0.001
A. caviae	25	6.2%	25	6.0%	32	6.0%	28	5.9%	35	5.0%	145	5.8%	0.404
A. sobria	26	6.5%	23	5.5%	29	5.5%	26	5.5%	25	3.6%	129	5.1%	0.029
Other*	14	3.5%	18	4.3%	17	3.2%	15	3.2%	20	2.9%	84	3.3%	0.575

Corynebacterium spp.	49	12.2%	73	17.6%	84	15.9%	95	20.1%	187	27.0%	488	19.4%	<0.001
C. striatum	15	3.7%	28	6.8%	37	7.0%	53	11.2%	132	19.0%	265	10.6%	<0.001
C. jeikeium	2	0.5%	5	1.2%	8	1.5%	10	2.1%	14	2.0%	39	1.6%	<0.001
C. afermentans	1	0.3%	2	0.5%	1	0.2%	5	1.1%	16	2.3%	25	1.0%	0.044
Other*	31	7.7%	38	9.2%	38	7.2%	27	5.7%	25	3.6%	159	6.3%	0.003
Micrococcus spp.	53	13.2%	49	11.8%	63	11.9%	24	5.1%	55	7.9%	244	9.7%	0.005
M. luteus	45	11.2%	45	10.8%	56	10.6%	24	5.1%	48	6.9%	218	8.7%	0.014
Other*	8	2.0%	4	01.0	7	1.3%	0	0.0%	7	1.0%	26	1.0%	0.176

				%									
Potential Bacterial Agents of Bioterrorism	22	5.5%	31	7.5%	25	4.7%	25	5.3%	65	9.4%	168	6.7%	0.022
Brucella spp.	16	4.0%	20	4.8%	11	2.1%	12	2.5%	42	6.1%	101	4.0%	0.142
Burkholderia pseudomallei	5	1.3%	10	2.4%	14	2.6%	12	2.5%	22	3.2%	63	2.5%	0.048
Other*	1	0.3%	1	0.2%	0	0.00 %	1	0.2%	1	0.1%	4	0.2%	1
Abiotrophia spp. and *Granulicatella*	22	5.5%	27	6.5%	41	7.7%	34	7.2%	41	5.9%	165	6.6%	0.773

spp.													
G. adiacens	14	3.5%	21	5.1%	31	5.9%	25	5.3%	28	4.0%	119	4.7%	0.652
A. defectiva	6	1.5%	6	1.5%	8	1.5%	8	1.7%	10	1.4%	38	1.5%	<0.001
Other*	2	0.5%	0	0.0%	2	0.4%	1	0.2%	3	0.4%	8	0.3%	0.149
Bacillus spp.	16	4.0%	22	5.3%	25	4.7%	28	5.9%	53	7.6%	144	5.7%	0.022
B. cereus	4	1.0%	13	3.1%	13	2.5%	21	4.5%	41	5.9%	92	3.7%	0.142
B. subtilis	10	2.5%	6	1.5%	6	1.1%	3	0.6%	2	0.3%	27	1.1%	0.048
Other*	2	0.5%	3	0.7%	6	1.1%	4	0.9%	10	1.4%	25	1.0%	0.694
Other*	61	15.2%	40	9.6%	74	14.0%	93	19.7%	102	14.7%	370	14.7%	0.818

*Some strains could not be identified as species, but only as genus, so they were classified as Other.

TABLE S9 Susceptibility of *Aeromonas* spp. to antimicrobial agents

Antimicrobial agent	*Aeromonas* spp. (n=252)				*A. hydrophila* (n=153)				*A. caviae* (n=39)				*A. sobria* (n=9)			
	No. of strain	R(%)	I(%)	S(%)	No. of strain	R(%)	I(%)	S(%)	No. of strain	R(%)	I(%)	S(%)	No. of strain	R(%)	I(%)	S(%)
CXM^{ND}	13	46.2	0.0	53.8	8	50.0	0.0	50.0	-	-	-	-	1	100.0	0.0	0.0
FOX^{ND}	13	46.2	7.7	46.2	9	55.6	11.1	33.3	-	-	-	-	1	100.0	0.0	0.0
CTX^{ND}	10	30.0	10	60.0	7	42.9	14.3	42.9	-	-	-	-	3	0.0	0.0	100.0
CTX^{NM}	151	19.2	0.7	80.2	85	17.6	1.2	81.2	30	33.3	0.0	66.7	-	-	-	-
CAZ^{ND}	23	26.1	0.0	73.9	16	25	0.0	75.0	1	0.0	0.0	100.0	-	-	-	-
IPM^{ND}	41	34.1	26.8	39.0	22	27.3	36.4	36.4	4	50	0.0	50.0	4	100.0	0.0	0.0
ATM^{ND}	19	21.1	0.0	78.9	12	16.7	0.0	83.3	1	0.0	0.0	100.0	-	-	-	-
AMK^{ND}	12	8.3	0.0	91.7	7	0.0	0.0	100.0	1	0.0	0.0	100.0	-	-	-	-

GENND	46	4.3	0.0	95.7	34	0.0	0.0	100.0	3	33.3	0	66.7	1	0.0	0.0	100.0
CIPND	11	0.0	18.2	81.8	7	57.1	14.3	28.6	1	0.0	0.0	100.0	-	-	-	-
TCYND	4	25.0	0.0	75.0	2	0.0	0.0	100.0	-	-	-	-	-	-	-	-
SXTND	8	50.0	0.0	50.0	5	60.0	0.0	40.0	-	-	-	-	-	-	-	-
CHLND	6	16.7	16.7	66.7	5	20.0	0.0	80.0	-	-	-	-	-	-	-	-

NM: microbroth dilution method; ND: The result of disk diffusion test methods; -: not measured;

TABLE S10 Susceptibility of *Corynebacterium* spp. to antimicrobial agents

Antimicrobial agent	*Corynebacterium* spp. （n=410）				*C. striatum* （n=206）				*C. jeikeium* （n=34）				*C. afermentans* （n=18）			
	No. of strain	R(%)	I(%)	S(%)	No. of strain	R(%)	I(%)	S(%)	No. of strain	R(%)	I(%)	S(%)	No. of strain	R(%)	I(%)	S(%)
PEN ND	213	82.2	0.0	17.8	88	94.3	0.0	5.7	20	90.0	0.0	10.0	9	77.8	0.0	22.2
PEN NM	115	53.0	33.9	13.0	70	61.4	30.0	8.6	9	66.7	11.1	22.2	3	100	0.0	0.0
CTX ND	86	58.1	8.1	33.7	32	81.2	6.2	12.5	8	25.0	25.0	50.0	3	100	0.0	0.0
CTX NM	30	70.0	3.3	26.7	18	88.9	0.0	11.1	1	100.0	0.0	0.0	-	-	-	-
VAN ND	274	0.0	0.0	100.0	123	0.0	0.0	100.0	25	0.0	0.0	100.0	11	0.0	0.0	100
VAN NM	120	0.0	0.0	100.0	78	0.0	0.0	100.0	7	0.0	0.0	100.0	5	0.0	0.0	100
GEN ND	165	26.7	5.5	67.9	73	27.4	9.6	63.0	20	35.0	0.0	65.0	9	33.3	11.1	55.6
ERY ND	208	67.8	23.6	8.7	77	74.0	26.0	0.0	16	68.8	25.0	6.2	11	90.9	9.1	0.0

CIP ND	206	84.0	1.9	14.1	90	95.6	2.2	2.2	19	78.9	0.0	21.1	13	84.6	0.0	15.4
DOX ND	23	4.3	0.0	95.7	10	10.0	0.0	90.0	5	0.0	0.0	100.0	2	0.0	0.0	100.0
TCY ND	162	13.0	5.6	81.5	78	14.1	6.4	79.5	15	13.3	0.0	86.7	9	0.0	11.1	88.9
CLI ND	272	86.4	7.4	6.2	121	92.6	5.8	1.7	23	82.6	8.7	8.7	12	91.7	8.3	0
SXT ND	151	55.6	9.9	34.4	54	59.3	7.4	33.3	13	76.9	7.7	15.4	6	66.7	0.0	33.3
RIF ND	155	22.6	0.6	76.8	61	1.6	1.6	96.7	11	9.1	0.0	90.9	9	66.7	0.0	33.3

NM: microbroth dilution method; ND: disk diffusion test methods; -: not measured;

TABLE S11 Susceptibility of *Micrococcus* spp. to antimicrobial agents

Antimicrobial agent	*Micrococcus* spp. （n=210）				*M. luteus* （n=189）			
	No. of strain	R(%)	I(%)	S(%)	No. of strain	R(%)	I(%)	S(%)
PEN [ND]	77	15.6	0.0	84.4	65	13.8	0.0	86.2
PEN [NM]	72	18.1	0.0	81.9	64	18.8	0.0	81.2
VAN [ND]	87	0.0	0.0	100.0	71	0.0	0.0	100.0
VAN [NM]	73	0.0	0.0	100.0	64	0.0	0.0	100.0
ERY [ND]	130	36.9	15.4	47.7	113	36.3	16.8	46.9
CLI [ND]	120	18.3	12.5	69.2	103	17.5	14.6	68

NM: microbroth dilution method; ND: disk diffusion test methods; -: not measured;

TABLE S12 Susceptibility of *Granulicatella* spp. and *Abiotrophia* spp. to antimicrobial agents

Antimicrobial agent	*Granulicatella* spp. *and Abiotrophia* spp. （n=139）				*Granulicatella adiacens* （n=99）				*Abiotrophia* spp. （n=40）			
	No. of strain	R(%)	I(%)	S(%)	No. of strain	R(%)	I(%)	S(%)	No. of strain	R(%)	I(%)	S(%)
PEN^{ND}	49	44.9	0.0	55.1	35	45.7	0.0	54.3	14	42.9	0.0	57.1
PEN^{NM}	47	2.1	29.8	68.1	35	2.9	31.5	65.7	12	0.0	33.3	66.6
PEN^{NE}	3	0.0	0.0	100.0	1	0.0	0.0	100.0	2	0.0	0.0	100.0
AMP^{ND}	42	2.4	0.0	97.6	26	3.8	0.0	96.2	16	0.0	0.0	100.0
CTX^{ND}	52	11.5	3.8	84.6	34	14.7	2.9	82.4	18	5.6	5.6	88.9
CTX^{NM}	7	0.0	0.0	100.0	6	0.0	0.0	100.0	1	0.0	0.0	100.0
IPM^{ND}	4	0.0	0.0	100.0	1	0.0	0.0	100.0	3	0.0	0.0	100.0

ERYND	119	58.8	10.1	31.1	83	59.0	10.8	30.1	36	58.3	8.3	33.3
CLIND	115	51.3	11.3	37.4	80	56.2	10.0	33.8	35	40.0	14.3	45.7
CIPND	7	14.3	14.3	71.4	1	0.0	100.0	0.0	6	0.0	0.0	100.0
CHLND	97	3.1	0.0	96.9	66	4.5	0.0	95.5	31	0.0	0.0	100.0
VANND	104	0.0	0.0	100.0	74	0.0	0.0	100.0	30	0.0	0.0	100.0
VAN^{NM}	16	0.0	0.0	100.0	10	0.0	0.0	100.0	6	0.0	0.0	100.0
VAN^{NE}	1	0.0	0.0	100.0	-	-	-	-	1	0.0	0.0	100.0

NM: microbroth dilution method; ND: disk diffusion test methods; NE: E-text; -: not measured;

TABLE S13 Susceptibility of *Bacillus* spp.to antimicrobial agents

Antimicrobial agent	*Bacillus* spp. (n=104)				*B. cereus* (n=63)				*B. subtilis* (n=25)			
	No. of strain	R(%)	I(%)	S(%)	No. of strain	R(%)	I(%)	S(%)	No. of strain	R(%)	I(%)	S(%)
PEN^{ND}	29	86.2	0.0	13.8	9	100.0	0.0	0.0	12	83.3	0.0	16.7
PEN^{NM}	47	38.3	31.9	29.8	36	88.9	0.0	11.1	6	100	0.0	0.0
PEN^{NE}	4	100.0	0.0	0.0	4	100.0	0.0	0.0	-	-	-	-
AMP^{ND}	22	81.8	4.5	13.6	14	78.6	7.1	14.3	4	100.0	0.0	0.0
IPM^{ND}	28	3.6	0	96.4	14	7.1	0.0	92.9	11	0.0	0.0	100.0
VAN^{ND}	46	0.0	0.0	100.0	21	0.0	0.0	100.0	17	0.0	0.0	100.0
VAN^{NM}	44	0.0	0.0	100.0	34	0.0	0.0	100.0	3	0.0	0.0	100.0
VAN^{NE}	4	0.0	0.0	100.0	4	0.0	0.0	100.0	-	-	-	-
AMK^{ND}	20	0.0	0.0	100.0	11	0.0	0.0	100.0	5	0.0	0.0	100.0

GEN^ND	36	0.0	2.8	97.2	19	0.0	0.0	100.0	14	0.0	7.1	92.9
ERY^ND	43	14	39.5	46.5	22	9.1	45.5	45.5	16	18.8	37.5	43.8
CLI^ND	45	15.6	66.7	17.8	20	5	70.0	25	17	11.8	76.5	11.8
TCY^ND	26	3.8	0	96.2	12	8.3	0.0	91.7	9	0.0	0.0	100.0
CIP^ND	33	15.2	51.5	33.3	20	20.0	55.0	25.0	9	0.0	11.1	88.9
SXT^ND	35	71.4	5.7	22.9	13	61.5	7.7	30.8	15	80.0	6.7	13.3
CHL^ND	26	3.8	3.8	92.3	18	0.0	5.6	94.4	6	16.7	0.0	83.3
RIF^ND	23	65.2	13	21.7	8	75	12.5	12.5	10	70.0	10.0	20.0

NM: microbroth dilution method; ND: disk diffusion test methods; NE: E-text; -: not measured;

TABLE S14 Susceptibility of Potential Bacterial Agents of Bioterrorism to antimicrobial agents

Antimicrobial agent	*Brucella* spp. （n=47）				Antimicrobial agent	*Burkholderia pseudomallei* （n=23）			
	No. of strain	R(%)	I(%)	S(%)		No. of strain	R(%)	I(%)	S(%)
SXT[ND]	1	0	0	100.0	SXT[ND]	11	45.5	18.2	36.4
GEN[ND]	45	0	0	100.0	AMC[ND]	3	100.0	0	0
DOX[ND]	1	0	0	100.0	CAZ[ND]	15	0	0	100.0
-	-	-	-	-	IPM[ND]	13	0	0	100.0
-	-	-	-	-	TCY[ND]	2	0	0	100.0
-	-	-	-	-	DOX[ND]	1	0	0	100.0

ND: the result of disk diffusion test methods; -: not measured;
